# A novel embryonic plasticity gene signature that predicts metastatic competence and clinical outcome

**DOI:** 10.1038/srep11766

**Published:** 2015-06-30

**Authors:** Rama Soundararajan, Anurag N. Paranjape, Valentin Barsan, Jeffrey T. Chang, Sendurai A. Mani

**Affiliations:** 1 Department of Translational Molecular Pathology; 2 Metastasis Research Center; 3 Center for Stem Cells and Developmental Biology, The University of Texas MD Anderson Cancer Center, Houston, Texas; 4 Department of Integrative Biology and Pharmacology, School of Medicine; School of Biomedical Informatics; The University of Texas Health Sciences Center at Houston, Houston, Texas

## Abstract

Currently, very few prognosticators accurately predict metastasis in cancer patients. In order to complete the metastatic cascade and successfully colonize distant sites, carcinoma cells undergo dynamic epithelial-mesenchymal-transition (EMT) and its reversal, mesenchymal-epithelial-transition (MET). While EMT-centric signatures correlate with response to therapy, they are unable to predict metastatic outcome. One reason is due to the wide range of transient phenotypes required for a tumor cell to disseminate and recreate a similar histology at distant sites. Since such dynamic cellular processes are also seen during embryo development (epithelial-like epiblast cells undergo transient EMT to generate the mesoderm, which eventually redifferentiates into epithelial tissues by MET), we sought to utilize this unique and highly conserved property of cellular plasticity to predict metastasis. Here we present the identification of a novel prognostic gene expression signature derived from mouse embryonic day 6.5 that is representative of extensive cellular plasticity, and predicts metastatic competence in human breast tumor cells. This signature may thus complement conventional clinical parameters to offer accurate prediction for outcome among multiple classes of breast cancer patients.

Much like the multi-step process of tumor progression to metastasis, mammalian embryo development encompasses an intricate series of morphogenetic events, and involves spatial and temporal coordination of multiple cell types as they proliferate, migrate, and differentiate to form complex higher-order organs and organ systems. The spatially-confined and sessile epithelial cells form tight cell-cell contacts in various organs, thus furnishing a critical barrier necessary to maintain a regulated environment. In contrast, the malleability offered by the motile and invasive mesenchymal cell type is what permits cell migration for tissue- and organ development. While these two cell states are quite distinct, they are also highly dynamic and interchangeable during embryo development, as well as during tumor progression to metastasis. A complex cellular reprogramming event called epithelial-to-mesenchymal-transition (or EMT) facilitates the conversion of differentiated epithelial cells (expressing surface E-cadherin) into loosely organized, highly migratory and invasive mesenchymal cells (lacking E-cadherin expression, among other profound alterations)^1^. Recent studies have established a strong molecular link between EMT and stem-cells, and further, suggested that EMT confers differentiated cells with stem-cell properties^2^,^3^. Because of the adaptive malleability that cancer cells acquire through the activation of EMT, many transcription factors (TF) capable of regulating EMT and their associated signaling pathways, have been proposed for the identification and classification of tumors that metastasize^4^,^5^,^6^. Supporting the importance of EMT in disease, the EMT inducer Snail has been shown to predict recurrence in breast cancer^7^.

Unfortunately, in many cases, gene expression profiles of EMT genes do not predict outcomes. For example, we previously generated an EMT-specific signature by over-expressing Snail or Twist or Goosecoid or TGFβ1 in breast epithelial cells^8^, but discovered that this signature was incapable of identifying patients who could potentially develop metastasis or tumor relapse (Suppl Figs 1,2 – column ‘EMT’). This suggests that EMT does not completely explain clinical outcomes. Other factors are also important. A solution to this conundrum came in a series of experiments that showed that mesenchymal cells that had undergone EMT are unable to form macrometastases^9^,^10^. The subsequent differentiation of these mesenchymal/stem-like cells, with restoration of epithelial features (the reverse process referred to as mesenchymal-to-epithelial-transition, MET), is what critically determines the formation of multiple tissues and organs during embryo development. It is becoming increasingly evident that the pathophysiological course of tumor cell invasion to metastasis is dependent *not* on the sole possession of particular EMT or MET or stem-cell traits, but instead on the innate flexibility of cancer cells in being able to dynamically switch between these various states, alter cellular morphology and function^1^,^11^,^12^. That is, metastatic cells must possess ‘plasticity’.

The past decade has witnessed the development of a number of gene expression signatures related to embryo development, cell migration, stemness or EMT, tested for use in cancer patient outcome predictions^13^,^14^,^15^,^16^,^17^,^18^,^19^,^20^,^21^,^22^. These comprise a wide variety of cell types of embryonic origin, lines that exhibit stem-cell features, cells which display classic EMT, and include different methods of EMT/stemness/pluripotency induction, or microarray/retrospective RT-PCR analyses of a defined set of cancer-related genes from patient samples. While these resources all have their own merit, they have either not been tested for metastasis predictions, or are unfortunately quite limited in their potential to accurately predict the metastatic recurrence of tumors or in their extended applicability across a broad spectrum of cancer subtypes.

Human breast cancer includes a highly diverse set of diseases, and is classified into at least 6 distinct clinically-relevant molecular subgroups - luminal A, luminal B, HER2+/ER-, basal-like, normal breast-like, and the most recently recognized claudin-low^23^,^24^. It is clear that at the molecular level, each breast cancer subtype displays distinct gene expression profiles, and exhibits significant differences in response to therapy and patient outcome^25^,^26^. Importantly, cancer cells with EMT- and stem-cell (CSC) properties are present in varying degrees among these different tumor subtypes, and may therefore not be adequately represented, in order to facilitate identification in gene signatures that are uniquely enriched for EMT/CSC traits. This is reiterated by the inability of our previously published EMT-signature to predict metastatic outcome^8^. This signature is, however, capable of identifying claudin-low tumors that are enriched with cells harboring mesenchymal and stem-cell traits, suggesting that EMT-centric signatures can only identify a “static cellular state” and not the “dynamic EMT/MET process”.

We therefore hypothesized that gene expression signatures that correlate with the time when the embryo undergoes extensive yet defined spatiotemporal cellular dynamics, that encapsulates a spectrum of changes that *together* permit cellular plasticity, would be superior to those rely on the expression of individual EMT/MET/stem-cell markers. In this manuscript, we document the discovery and characterization of one such unique gene expression signature derived from mouse embryo development (E6.5), that can forecast breast cancer patient survival rate. We further describe a novel score that can be used to quantify the extent of cellular plasticity in the context of breast tumor progression, and demonstrate that this score, which is based on the E6.5 signature, can accurately predict the metastatic ability of tumor cells.

## Results

### Derivation of a physiologically-relevant embryonic gene expression signature that reflects cellular plasticity

The parallels observed in cellular behavior during early embryo development and tumor metastasis support the hypothesis that metastatic competence in cancer cells is dictated by cellular plasticity, which can be modeled by examining similar gene expression patterns during embryogenesis. We therefore sought to isolate a specific developmental stage demonstrative of high cellular plasticity, and utilize its gene expression profile as a unique tool to predict tumor metastasis.

**Figure 1:**
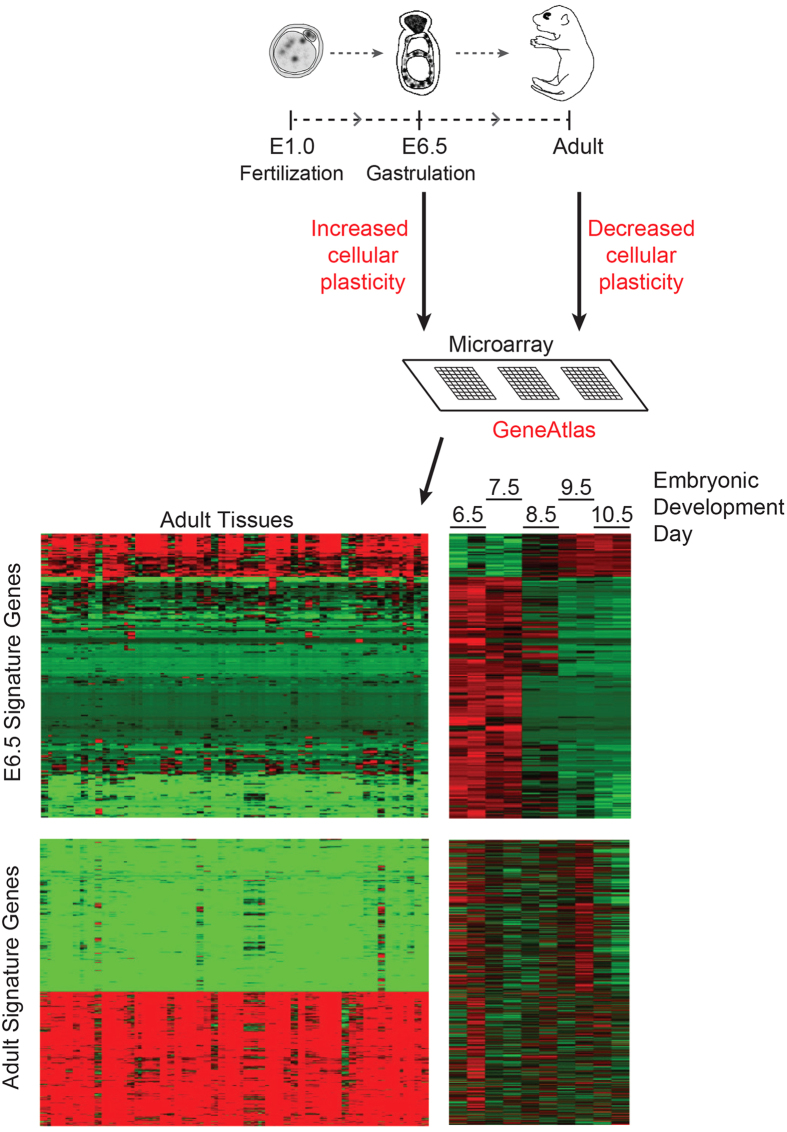
Derivation of a stage-specific gene expression signature from mouse embryonic development, as well as from adult murine tissues. A three-step schematic illustrating the generation of gene signatures specifically from murine E6.5- and adult tissues using the published Gene Atlas microarray database^28^. The heat maps show the expression profile of genes in these signatures. The left column represents the expression of the genes in adult tissues and the right column shows expression at various stages of mouse embryonic development (E6.5–E10.5), with green indicating lower gene expression and red representing higher relative expression. Each row represents a gene, and each column, a specific tissue.

Among the various stages of murine embryonic development, the onset of gastrulation occurs at E6.5 and represents high cellular plasticity^11^,^27^. Gastrulation begins with the establishment of the ‘primitive-streak’, which provides a path for future cell migration. Cells from the epiblast migrate into the core of the embryo *via* this ‘primitive-streak’, in a process termed ingression, which involves EMT. The event of EMT bestows early epithelial cells of the epiblast with the flexibility associated with a more invasive and motile phenotype, required for movement along, and eventually away from, the ‘primitive-streak’ to define the trilaminar disc comprised of three germ layers (ectoderm, mesoderm and endoderm) that eventually contribute to the formation of the various organs^11^,^27^. This process of trilaminar cell allocation is completed by E8.0. E6.5 of the developing mouse embryo is thus representative of extensive cellular plasticity, wherein epithelial cells are programmed to traverse the streak. We therefore analyzed the expression of genes at E6.5 of murine embryonic development, using the published database at the BioGPS website^28^, and thus arrived at a gene signature, which is representative of cellular plasticity. We further reasoned that as a corollary, a “differentiated” expression signature identified from “adult” tissues, which are not expressed in the embryo, should serve as the corresponding negative correlate representative of decreased cellular plasticity. Figure 1 illustrates the source and step-wise derivation of the E6.5- and adult gene expression signatures. The complete list of genes constituting both signatures is provided in Suppl Table-1.

### Characterization of the E6.5 plasticity gene signature

**Figure 2:**
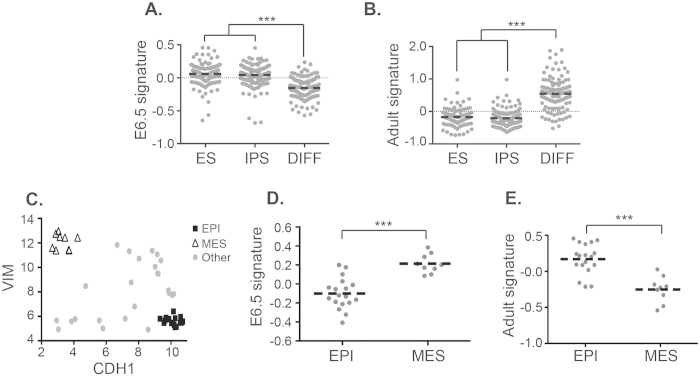
The newly identified E6.5 gene signature recognizes features representative of the mesenchymal phenotype, embryonic stem-cells (ES), as well as induced pluripotent stem-cells (IPS). Scatter plots **A** and **B** show that the E6.5 signature identifies with ES- and IPS- gene expression profiles procured from publicly available cell line databases, whereas the adult signature (negative correlate) recognizes the profile of the differentiated phenotype. Each dot represents a distinct cell line. Next, numerous cancer cell lines from the E-TABM-157 database were segregated as epithelial (EPI) or mesenchymal (MES), based on their expression levels of E-cadherin (CDH1) and Vimentin (VIM), and the “strongly epithelial (squares)” and “strongly mesenchymal (triangles)” sources alone (**C**) were used to generate comparative gene expression profiles for scoring the newly identified signature. Scatter plot **D** demonstrates that the E6.5- signature distinctly correlates with the EMT archetype, strongly recognizing mesenchymal profiles of breast cancer cell lines, while the adult signature does not (**E**). ***p < 0.001, ES-Embryonic Stem cell signature, IPS- Induced pluripotent stem cell signature, DIFF- Differentiated cell signature.

Because of the significantly increased expression of EMT- and stem cell-related markers at this stage of embryonic development, we investigated the ability of the newly identified signature to recognize features characteristic of stem cells, as well as cells displaying the classic EMT phenotype. Figure 2A demonstrates the correlation of the E6.5 signature with embryonic stem cell- and induced pluripotent stem cell-gene expression profiles obtained from publicly available gene expression databases. In contrast, the adult signature (the intended negative correlate) recognizes the profile of the differentiated phenotype (Fig. 2B). We further confirmed the potential of the E6.5 signature for recognition of the EMT archetype in a comprehensive cancer cell line database (E-TABM-157) (Fig. 2C–E).

### A quantitative score to predict the metastatic behavior of breast tumor cells

**Figure 3:**
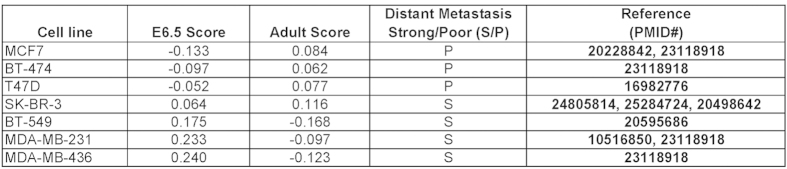
A “plasticity” score conferred by the E6.5 signature accurately predicts the metastatic competence of breast tumor cells. Various human breast cancer cell lines of known distant metastatic capacity *in vivo* were scored for their concordance with the E6.5- or adult-signature, and assigned a ‘plasticity’ score. A positive E6.5-score correlated strongly with the propensity for distant metastasis, and a negative score, poorly. In contrast, a positive adult-score correlated negatively with the propensity for distant metastasis (also refer to Suppl Fig. 3 for a quantitative representation of assessment of metastatic competence).

The genes defining the E6.5 signature exhibit remarkable functional diversity ranging from cellular maintenance, tissue morphology and embryonic/organismal development to lipid metabolism, free radical scavenging, and cancer metastasis (Suppl Table-2), the sum total of which, we reasoned, should collectively exemplify cellular plasticity. We next compared various human breast tumor cell lines of established metastatic capacity in animal models, and scored them based on their expression of the E6.5 signature (Fig. 3). Interestingly, a positive E6.5 score consistently correlated with a strong propensity for distant metastasis, while a negative score suggested lack of ability to metastasize (Fig. 3, Suppl Fig. 3A). As expected, and in contrast, a positive adult score correlated with an increased propensity for distant metastasis (Fig. 3, Suppl Fig. 3B).

### Established EMT factors fail in their ability to reliably predict distant metastasis or tumor recurrence

**Figure 4:**
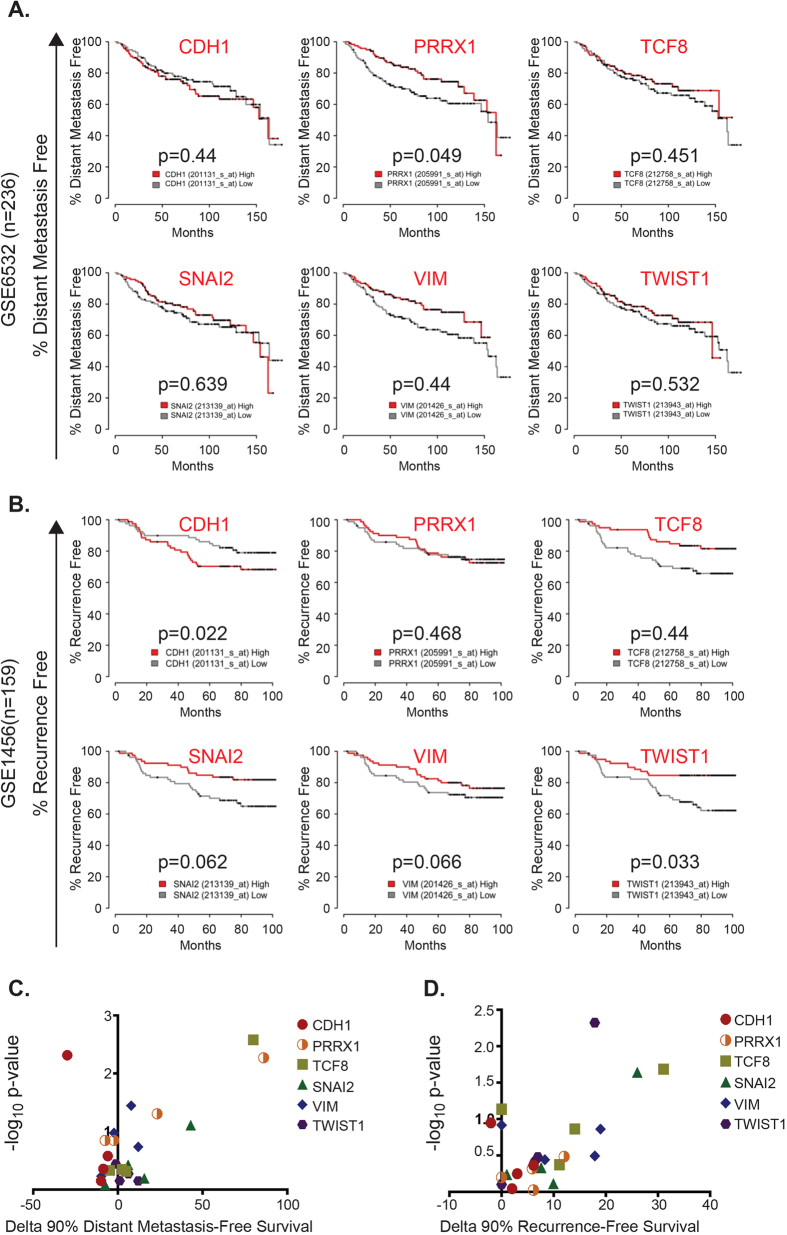
Classic EMT-/stem cell-related markers are poor predictors of distant metastasis-free survival (DMFS) as well as recurrence-free survival (RFS) in breast cancer patients. Patient cohorts representing various subtypes of breast cancer were discretized into high- or low-expression groups, based on their concordance with expression of well-established EMT-/stem-cell markers popularly tested for risk assessment (full gene-list provided in Suppl Fig. 8A; also refer to compiled Kaplan-Meier prediction data in Suppl Figs 8B, C). Panel **A** shows DMFS curves for 6 well-known EMT-/stem cell-related factors, based on data analyzed from one patient cohort (GSE6532). Cumulative data from survival curves of all four patient cohorts analyzed (represented as 4 distinct colored dots; also refer to individual Kaplan-Meier plots in Suppl Figs 4, 5) were aligned on a scatter plot, and their performance in foretelling risk for distant metastasis was tested in C. Panel B shows respective representative RFS curves based on data analyzed from one patient cohort (GSE1456). Cumulative data from survival curves of all four patient cohorts analyzed (represented as 4 distinct colored dots; also refer to individual Kaplan-Meier plots in Suppl Fig. 6, 7) were aligned on a scatter plot, and their performance in foretelling risk for tumor recurrence was tested in D. CDH1: E-cadherin; PRRX1: Paired mesoderm homeobox 1; TCF8: Zinc finger E-box-binding homeobox 1 or Zeb1; SNAI2: Snail2 or Slug; VIM: Vimentin; TWIST1: Twist1.

We next tested the predictive power of selected well-recognized EMT- or stem cell-related genes, in foretelling distant metastasis and tumor recurrence among breast cancer patients, in four independent publicly available databases [GSE20685, GSE6532_GPL96, GSE7390, GSE11121 for distant metastasis-free survival (DMFS) analyses, and GSE1456, GSE12276, GSE4922_UPPSALA, and GSE21653 for recurrence-free survival (RFS) analyses]. Representative DMFS- and RFS analyses using 6 key EMT- or stem cell-related genes (CDH1, PRRX1, TCF8, SNAI2, VIM and TWIST1), from one cohort, are shown in Fig. 4A,B respectively. Cumulative analyses from all four cohorts (displayed as 4 distinct colored dots, each dot representing one cohort) for these 6 genes, are presented in Fig. 4C,D. A more detailed analysis querying the relevance of 39 well-recognized EMT- or stem cell-related genes (in all 4 cohorts), is presented in Suppl Fig. 8.

As clearly discernable in Fig. 4 (and Suppl Fig. 8), most classic EMT- or stem cell-related markers considered individually, are poorly prognostic. They are mostly either non-predictive, or predict the opposite result. Further, their performance is inconsistent across the datasets. E-cadherin (“purple” dots) is a good example. E-cadherin is perhaps the most commonly used determinant of the EMT phenotype. E-cadherin is an epithelial cell adhesion molecule, and its loss has been reported to be a prerequisite for the onset of EMT and tumor cell migration^29^. However, our results demonstrate its poor prognostic power for determination of DMFS (Fig. 4A-CDH1; Fig. 4C-“purple” dots) and RFS (Fig. 4B-CDH1; Fig. 4D-“purple” dots), and further, indicate its inconsistency when tested in multiple patient datasets (represented as 4 distinct “purple” dots, Fig. 4C,D).

### The E6.5 gene signature has prognostic value in predicting cl**i**nical outcome

**Figure 5:**
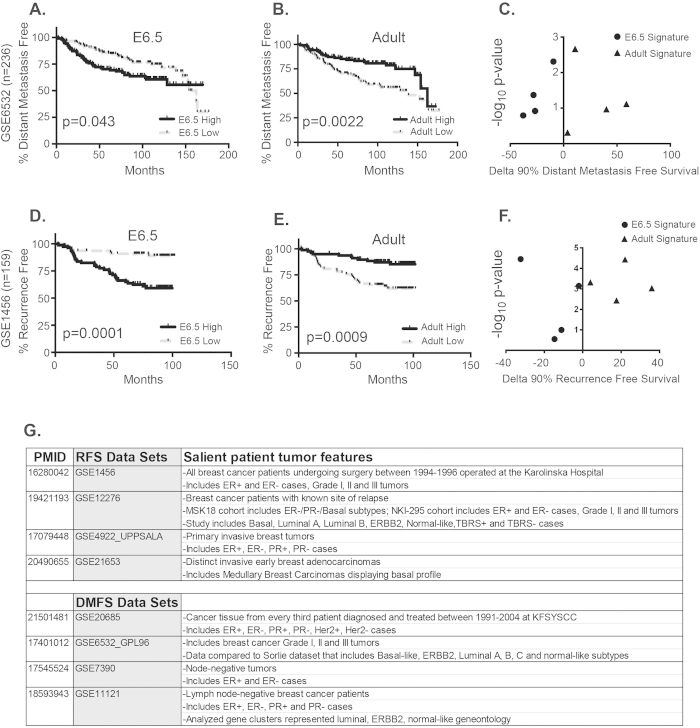
The E6.5 gene expression signature reflecting the plasticity phenotype, predicts distant metastasis-free survival (DMFS) as well as recurrence-free survival (RFS) in breast cancer patients. Patients cohorts representing various subtypes of breast cancer were discretized into high- or low-expression groups, based on their concordance with E6.5, or adult signatures. **A** and **B** show respective representative DMFS curves based on data analyzed from one patient cohort (GSE6532). Cumulative data from survival curves of all four patient cohorts analyzed (represented as 4 distinct dots; also refer to individual Kaplan-Meier plots in Suppl Fig. 1) were aligned on a scatter plot, and their performance in foretelling risk for distant metastasis was validated in **C**. **D** and **E** show respective representative RFS curves based on data analyzed from one patient cohort (GSE1456). Cumulative data from survival curves of all four patient cohorts analyzed (represented as 4 distinct dots; also refer to individual Kaplan-Meier plots in Suppl Fig. 2) were aligned on a scatter plot, and their performance in foretelling risk for tumor recurrence was validated in **F**. As expected, the adult signature (triangles) correlates with better survival in both analyses. The identity of various publicly available patient databases used for these analyses is listed in **G**, along with the included breast tumor types in each dataset. ER: Estrogen receptor; PR: Progesterone receptor; ERBB2/Her2: Human epidermal growth factor receptor 2; TBRS: TGFβ-responsive signature.

Since the identified gene signature from E6.5 is reflective of cellular plasticity, a property critical for carcinoma metastasis, we next queried if the net pattern of gene expression at this particular stage of the developing mammalian embryo, can be used for prognosis of DMFS and RFS, in all four datasets. Representative graphs from one cohort are presented in Fig. 5A,D (Individual survival graphs for DMFS and RFS, across all cohorts tested, are provided in Suppl Figs 1 and 2 respectively – “E6.5”). We observed that the E6.5 signature was capable of accurately stratifying patients according to risk for distant metastasis as well as for tumor recurrence (Fig. 5C,F; each database is represented as a distinct shape in each plot).

In contrast, and as expected, the adult signature (triangles) representative of decreased cellular plasticity, served as the negative correlate for the clinical prediction. Patients exhibiting a “high” score for the “adult” signature clearly demonstrated better clinical outcomes, in independent patient cohorts (represented as 4 separate triangles) (Fig. 5B,C,E,F, Suppl Figs 1 and 2 – “Adult”).

Notably, the datasets used for DMFS and RFS analyses comprised heterogeneous groups of breast cancer patients that were assigned to various molecular sub-categories. The specific tumor subtypes included in each study are highlighted in Fig. 5G. These data suggest that the newly identified E6.5 signature captures a distinct gene expression pattern that is independently indicative of tumor recurrence and/or distant metastasis among a wide array of breast cancer patients - luminal A, -B, -C, ER+/−, PR+/−, Her2+/−, basal and grade I, II or III tumors.

## Discussion

EMT is often heralded as a significant participant in tumor progression, and many studies have provided morphological evidence for EMT at the invasive fronts of human tumors^30^,^31^. Interestingly however, the EMT status of the primary tumor does not independently predict post-operative recurrence or disease-free survival in cancer patients^18^,^32^. Although EMT-endowed migratory and invasive properties contribute to the dissemination of carcinoma cells, in many instances, these features *alone*, are not sufficient for carcinoma cells to complete the cascade leading to eventual macrometastatic colonization. Recent work has highlighted the necessity for down-regulating potent EMT-inducers such as Snail, Prrx1 or Twist, in order to allow for distant metastasis^9^,^10^,^33^. Thus constitutive/sustained/unregulated EMT actually *suppresses* metastasis. In support of this notion, Ocana *et al*., found no clear correlation between the expression of Twist or Snail in primary tumors and relapse-free survival in breast cancer patients^9^. Moreover, carcinoma macrometastases at distant sites share an epithelial histopathology similar to that of the primary tumor at the site of origin^10^,^34^,^35^. While this appears paradoxical, and in a way, questions the participation and relevance of EMT in cancer progression, the following outlook lends a plausible explanation, accounting for both sets of observations^9^,^10^,^33^,^36^,^37^ - EMT is a transitory phenomenon employed by carcinoma cells during their multistep progression towards metastasis. Such migratory cancer cells harboring increased expression and function of classical EMT-TFs invade through the extracellular matrix, enter the blood and lymphatic vessels, and thereby spread to new locations within the body. While EMT- and stem cell-related traits are prominent players in the initial stages of metastasis, loss of these mesenchymal/stem-cell attributes and simultaneous retrieval of epithelial features (namely, MET) is what permits the establishment of metastatic colonies at outlying sites. These distant tumors are therefore still epithelial (hence, similar to the primary tumors); however, they have additionally recovered the proliferative state necessary for subsistence.

This implies that cells that are able to successfully chart the EMT-MET course (presumably in response to various intrinsic and extrinsic factors) have a higher propensity to metastasize. In other words, it is the plasticity of the carcinoma cell that dictates whether or not it can metastasize. Implicit in this notion, is the fact that gene expression signatures that are solely based on EMT (in other words, signatures representing features required for completing just one-half of the journey to metastasis), therefore cannot reliably predict distant colonization. We therefore reasoned that plasticity-bestowing traits have to be necessarily factored into this equation, in order to be able to foretell metastatic competence.

Interestingly, although whole-tumor gene expression profiles are quite distinct across the various molecular subtypes of human breast cancer, the stem-cell populations isolated from these tumors (that are considered to be the harbingers of metastases), exhibit high conservation in gene expression patterns, that is independent of the sub-classification^38^. Importantly, these cancer stem-cells were shown to readily transition between epithelial and mesenchymal states^38^. This further emphasized that a common biological phenomenon such as “cellular plasticity” (that can be conserved across the various subtypes) would better serve as the basis for the development of gene expression signatures aimed at impartially addressing all breast tumors.

The E6.5 signature presented in this manuscript in unique in that it denotes a net gene expression pattern indicative of “cellular plasticity” (regardless of the expression states of individual EMT/stem-cell/pluripotency markers), and thus is capable of reliably predicting DMFS and RFS in breast cancer patients, better than some of the most well-recognized EMT/stem-cell genes considered individually (Figs 4 and 5). While the E6.5 signature does indeed recognize features characteristic of stem-cells and the EMT phenotype, included in this signature list are various other protein kinases, proliferation modulators and regulators of lipid metabolism, among others (Suppl Tables-1, 2), the sum total of which is predictive of tumor spread. This discovery complements recent work involving other cancers (lung) that demonstrate that acquisition of ectopic embryonic gene expression profiles characterizes tumors of the aggressive phenotype^39^. Indeed, assignment of a score based on concordance with the E6.5 signature (which we have termed the “plasticity score”) to some of the most popular human cell lines used for breast cancer research (Fig. 3), or even prostate cancer research for that matter (Suppl Fig. 9), appears to separate them based on their ability to invade and metastasize *in vivo*, with a striking degree of accuracy.

### Prognostic value of the E6.5 embryonic gene signature

Histopathological grading is still the most commonly used clinical prognostic indicator for breast cancer patients, and the vital benchmark for the appraisal of therapeutic options. This evaluation is, however, based on anatomical information, or the binary presence/absence of a few select markers. Existing biomarkers are limited in their application because breast cancer is a heterogeneous disease, and patients often exhibit drastic differences in response to therapy despite similarities in histological types, grade and stage. For example, while analysis of E-cadherin expression may be helpful as an aid to the histopathological sub-classification of breast tumors^40^, our study demonstrates that its practical utility as a prognostic biomarker is questionable because reproducibility across multiple datasets is an apparent problem (Fig. 4). Although many studies suggest a direct association between reduced E-cadherin expression and high histopathologic grade, its correlation with metastatic progression is far less consistent^41^,^42^,^43^. Further, E-cadherin expression has been clearly shown to have no prognostic role in the intermediate stage II cases of breast cancer^44^ (which represent 30–60% of all cases, and are also the major source of inter-observer discrepancy among pathologists, making treatment decisions difficult), thus limiting its potential use as a common predictive factor applicable to all breast tumors.

Two well-characterized gene expression signatures for breast cancer that have been translated to the clinic are the Mammaprint (a 70-gene signature^45^,^46^) and the Oncotype, (a 21-gene signature^14^). While their reproducibility has been tested in multiple independent patient cohorts across the world, their use is unfortunately limited to patients with ER-positive disease. Similarly, the 241-gene signature capable of isolating patients at risk for late relapse, identified by Mittempergher *et al*.^47^, is designed for ER-positive/HER2-negative patients. The prognostic power of the 186 gene-based “invasiveness gene signature” developed by Liu *et al*.^48^ (derived from genes differentially-expressed in tumorigenic CD44^hi^/CD24^lo^ breast cancer cells), which is associated with increased risk of metastasis and early death, is significant only in patients with ER-positive and intermediate grade tumors. On the other hand, a recently described 41-gene signature derived from breast cancer stem-cells, or a 31-gene signature derived from tumor-initiating stem-like cells that demonstrated good prognostic significance with a strong capability of predicting distant metastasis-free survival and overall-survival, were only able to perform well in ER-negative breast cancer patients^17^,^49^.

In contrast, our results indicate that the E6.5 gene signature, that addresses a common biologic property underlying tumor spread among most sub-classifications of breast cancer, is able to serve as a reliable prognostic predictor of distant metastasis and poor clinical outcome among a broad range of patients (Fig. 5).

**Figure 6:**
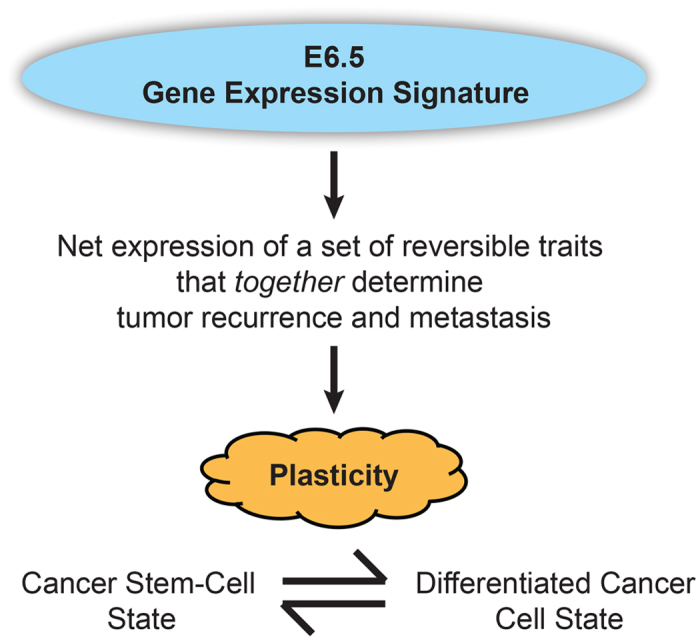
Study summary schematic. The newly identified E6.5 gene expression signature derived from a specific stage of the developing murine embryo, which is representative of high cellular plasticity (and reflective of the potential to readily switch between distinct cell states), encapsulates a spectrum of changes that, *collectively*, recognizes the ability of breast tumors to relapse and/or metastasize to distant organs.

**In conclusion**, we document the identification and potential application of a unique gene expression signature derived from the E6.5 stage of mouse embryonic development (Fig. 6), as a reliable prognostic indicator that may guide treatment options in breast cancer patients based on estimated survival benefit, as well as aid in monitoring of disease progression. This signature is unique in that it encapsulates a distinct biologic property that appears to dictate the metastatic competence of tumor cells, and because its prognostic power is applicable to a wide range of breast cancer patients. Further, the E6.5 score can be applied to predict the metastatic capacity of breast tumor cells *in vivo*. Directed prospective studies in the future will determine if this signature can indeed complement classic prognostic factors to improve patient outcome.

## Methods

### Derivation of the E6.5- and adult gene expression signatures

We downloaded the GeneAtlas gene expression data, as well as the accompanying annotations from the BioGPS website^28^, and converted the mouse Entrez Gene IDs to human using the Homologene database^50^. We then excluded the samples from cell lines, those that had been stimulated, or those from undeveloped tissue (excluding tissues from embryonic day 6.5 to 10.5), and logged expression values with base 2.

To generate the embryonic day 6.5 signature, we compared the expression at E6.5 and E7.5 against those at E9.5, E10.5, and adult tissues. We omitted day E8.5 because, based on the expression patterns, it appeared to be a transition period between the earlier and later time points. We selected the genes that were differentially expressed with a 5-fold change and false discovery rate <0.05, as determined by an empirical Bayes approach^51^. This identified 190 probes that were expressed significantly higher in embryonic days 6.5 and 7.5, and 34 probes higher in day E9.5, E10.5, and adult tissues. For the differentiated signature, we compared the expression in adult tissues against those at E6.5, E7.5, as well as E9.5 and E10.5. We selected the genes for this signature using the same strategy, resulting in 370 probes higher in adult tissues, and 420 probes higher in embryonic days 6.5, 7.5, 9.5, and 10.5. Significantly differentially up-regulated (“Up”) and down-regulated (“Down”) genes were combined in each set to develop a unique stage-specific signature. Complete gene list is presented in Suppl Table-1.

To score the signatures on a gene expression data set, we first processed it. If an Entrez Gene ID matched multiple probes on the array, we selected the probe with the highest variance across the data set. Then, for each gene, we normalized it so that the mean and variance would be 0 and 1, respectively. To calculate the score for a signature, we averaged the expression values for the genes in the signature after applying a weight of 1 for the up-regulated genes and −1 for the down-regulated ones. This yielded a signature score for each sample in the data set.

### Patient gene expression- and clinical data

We obtained the raw CEL files, together with patients’ characteristics, from the following public databases - GSE20685^52^, GSE6532_GPL96^53^, GSE7390^54^, GSE11121^55^, GSE1456^56^, GSE12276^57^, GSE4922_UPPSALA^58^, and GSE21653^59^. We then preprocessed the CEL files with RMA^60^.

### Survival analysis

Outcome data included recurrence-free survival (RFS) and distant metastasis-free survival (DMFS) analyses. Kaplan-Meier plots were used for the survival analyses. To quantify the differences in outcome between two groups of patients, we compared the differences in time before 10% of the patients acquired the outcome (either RFS or DMFS), because of the relatively low frequency of these events in cancer data sets. We calculated the p-values using a log rank test.

### Gene network enrichment analyses

QIAGEN’s Ingenuity® Pathway Analysis (IPA®, QIAGEN Redwood City, www.qiagen.com/ingenuity) was used to identify enriched functional gene networks among differentially regulated transcripts in the E6.5 signature. The full gene list (Suppl Table-1) that resulted from the significance analysis of the microarrays was used for the IPA analysis. P-values were calculated by IPA using a right-tailed Fisher Exact test (a cutoff of p < 0.05 was considered significant).

## Additional Information

**How to cite this article**: Soundararajan, R. *et al*. A novel embryonic plasticity gene signature that predicts metastatic competence and clinical outcome. *Sci. Rep*. **5**, 11766; doi: 10.1038/srep11766 (2015).

## Supplementary Material

Supplementary Information
