# Supplementary material for: A novel embryonic plasticity gene signature that predicts metastatic competence and clinical outcome
Source: Sci Rep. 2015 Jun 30;5:11766. doi: 10.1038/srep11766 (PMC4485318; doi:10.1038/srep11766)
Supplement: Supplementary Information [file srep11766-s1.pdf]

A novel embryonic plasticity gene signature that predicts metastatic competence and clinical outcome

Rama Soundararajan[1], Anurag N. Paranjape[1], Valentin Barsan[1], Jeffrey T. Chang[4,*] and Sendurai A. Mani[1,2,3,*]

[1]Department of Translational Molecular Pathology, [2]Metastasis Research Center, [3]Center for Stem Cells and Developmental Biology, The University of Texas MD Anderson Cancer Center, Houston, Texas.

[4]Department of Integrative Biology and Pharmacology, School of Medicine; School of Biomedical Informatics; The University of Texas Health Sciences Center at Houston, Houston, Texas.

[*]**Co-corresponding authors:** Jeffrey T. Chang, [4]Department of Integrative Biology and Pharmacology, School of Medicine; School of Biomedical Informatics; The University of Texas Health Sciences Center at Houston, Houston, Texas. E-mail: Jeffrey.T.Chang@uth.tmc.edu; Sendurai A. Mani, Department of Translational Molecular Pathology, Metastasis Research center, Center for Stem Cells and Developmental Biology, The University of Texas MD Anderson Cancer Center, Houston, Texas 77030. E-mail: mani@mdanderson.org.

Running title: Employing cellular plasticity to determine metastatic competence

Suppl Figure. 1

E6.5/Adult/EMT-signatures, Survival Curves, Distant Metastasis-Free Survival

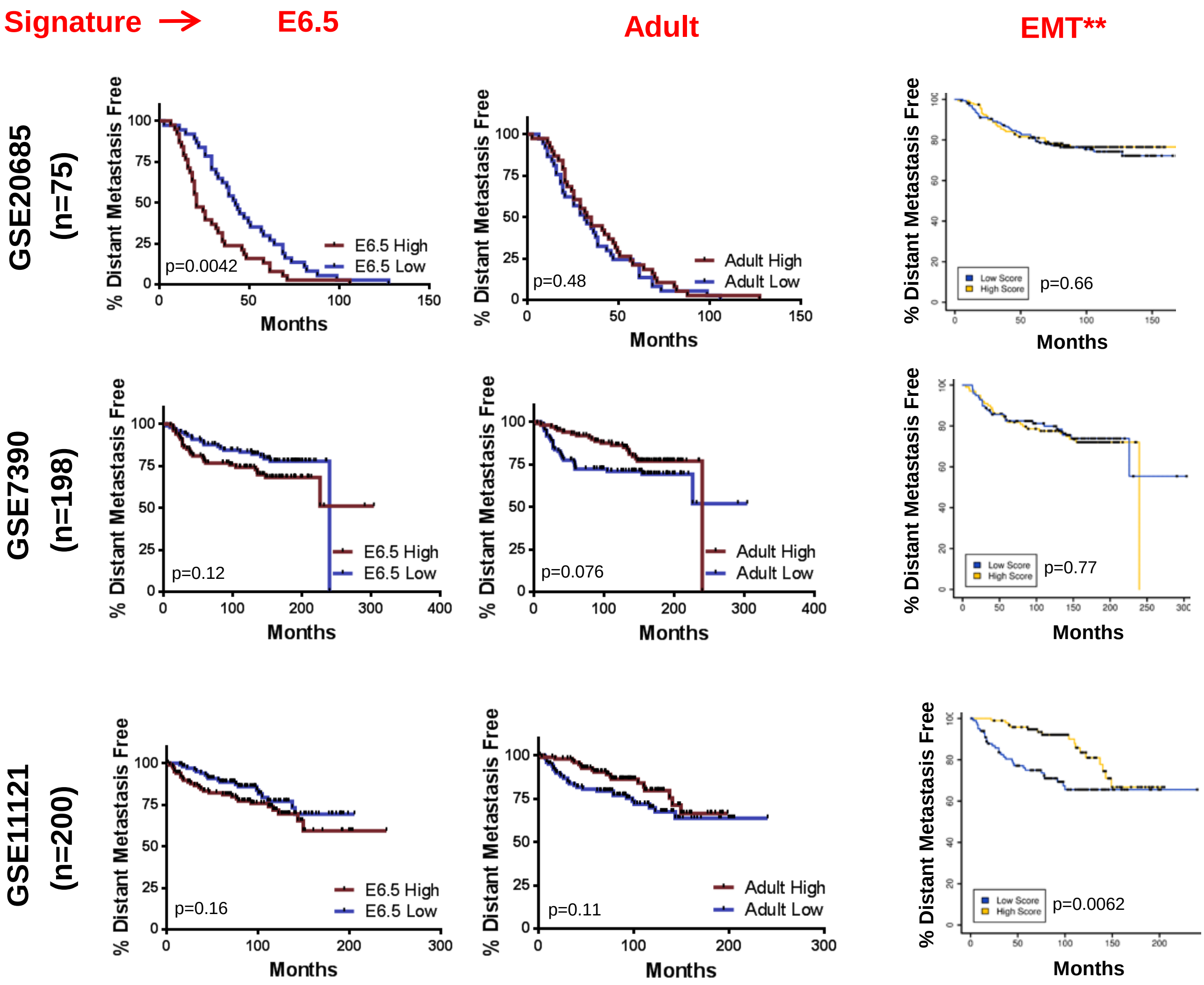

Our previously-published EMT signature:

[Taube *et. al.,* PNAS 2010 Aug 31;107(35):15449-54]

Suppl Figure. 2

E6.5/Adult/EMT-signatures, Survival Curves, Recurrence-Free Survival

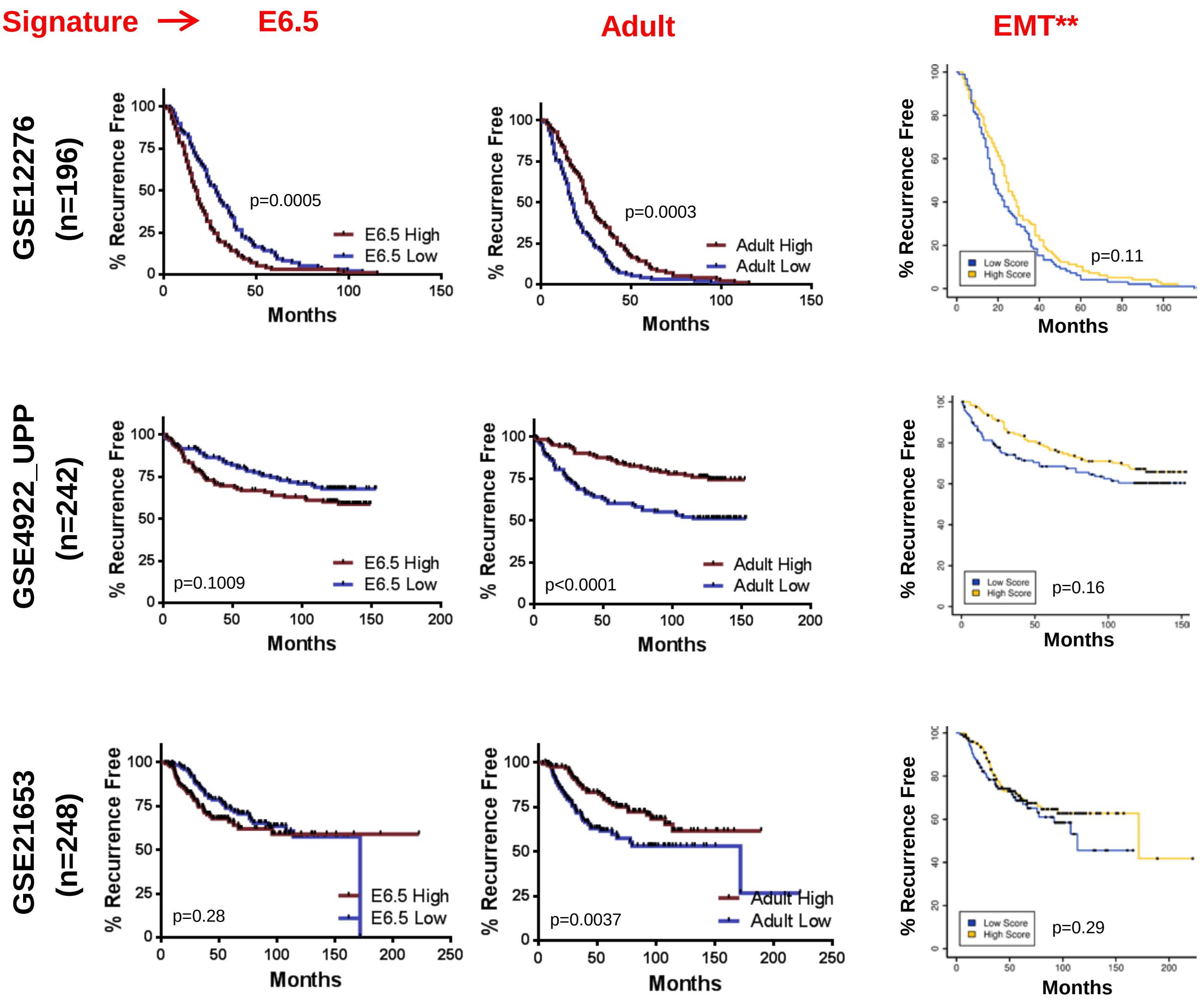

Our previously-published EMT signature:

[Taube *et. al.,* PNAS 2010 Aug 31;107(35):15449-54]

Suppl Figure. 3

Determining metastatic competence of commonly used breast cancer cell lines based on concordance with E6.5- or Adult gene expression signatures.

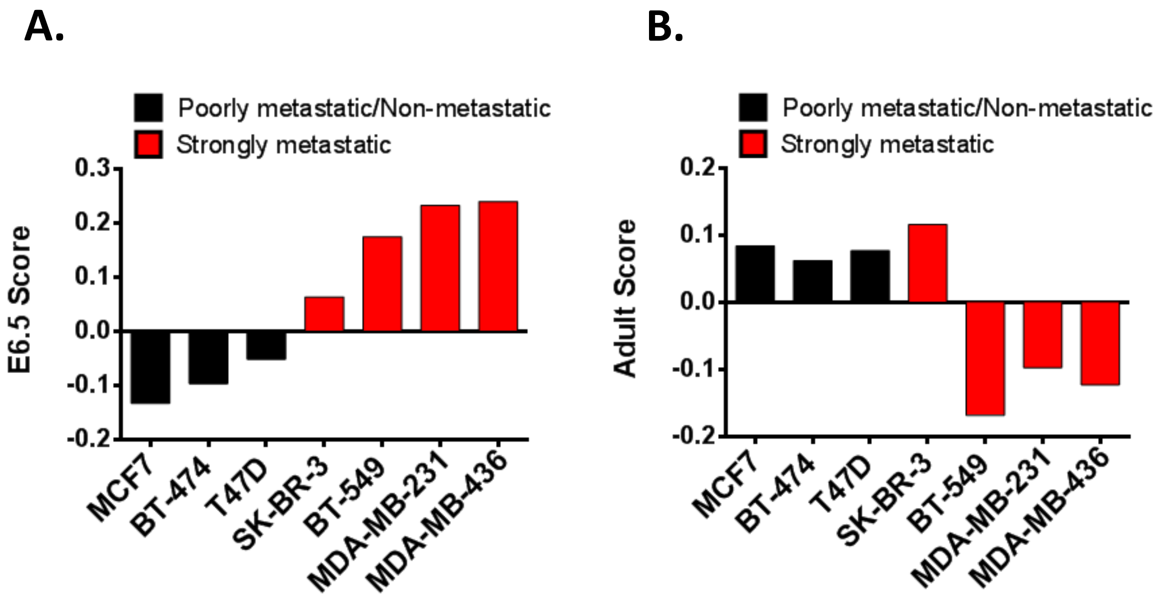

Suppl Figure. 4

Key EMT-related Factors, Survival Curves,
Distant Metastasis-Free Survival –Set 1

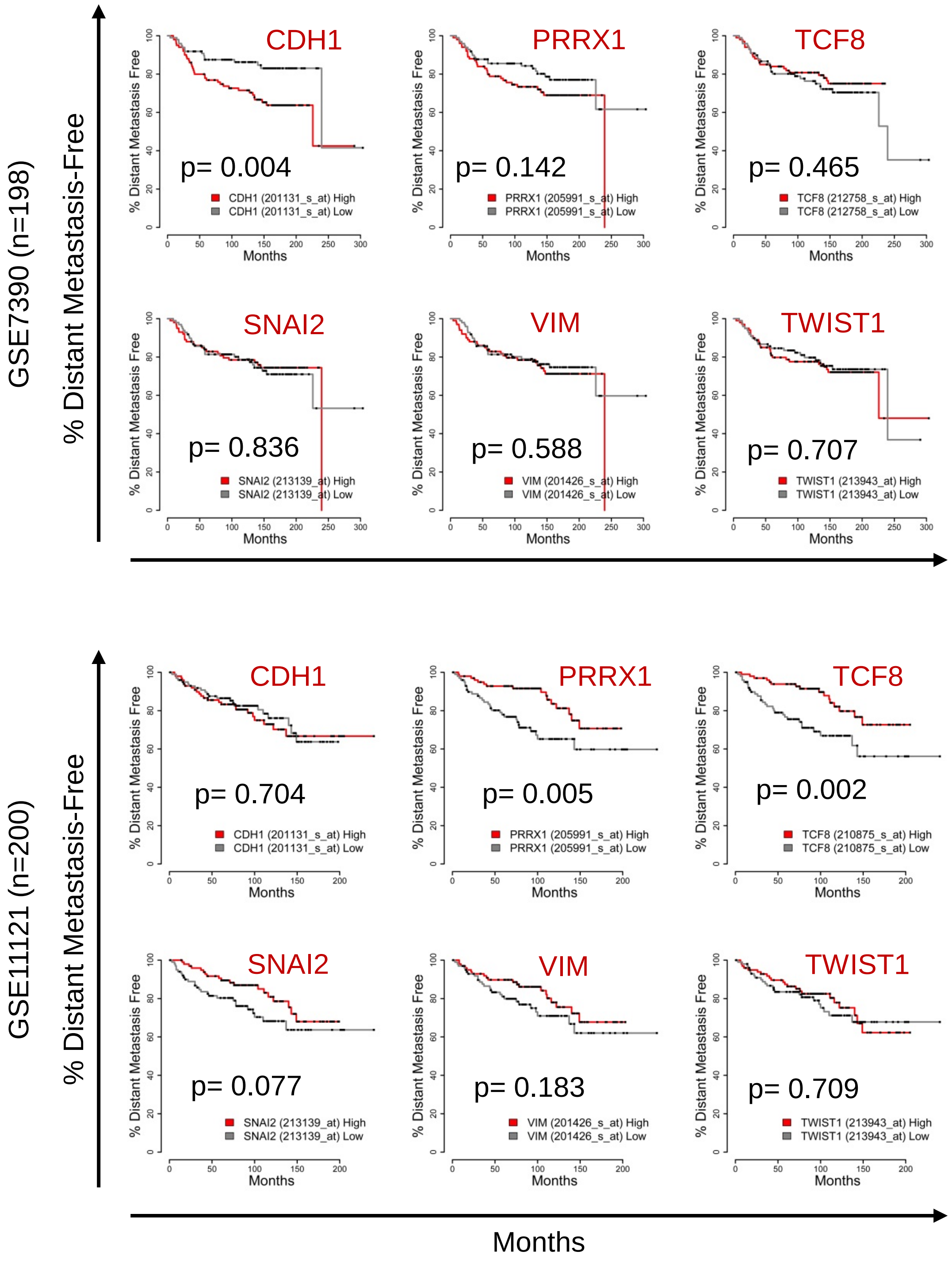

Suppl Figure. 5

Key EMT-related Factors, Survival Curves,
Distant Metastasis-Free Survival –Set 2

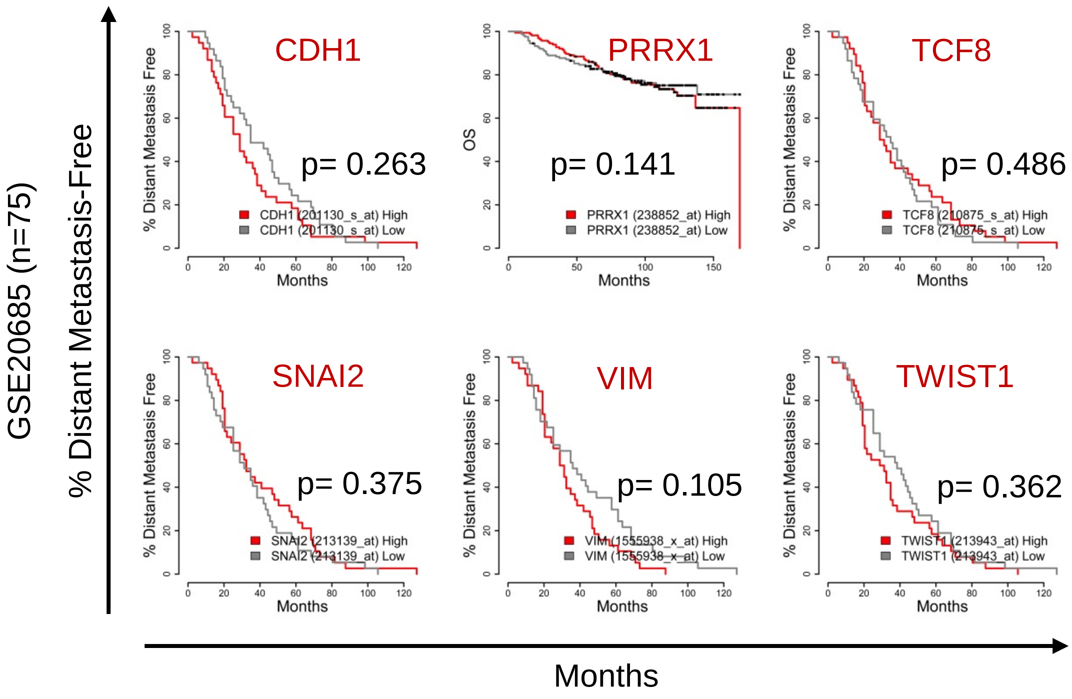

Suppl Figure. 6

Key EMT-related Factors, Survival Curves,
Recurrence-Free Survival –Set 1

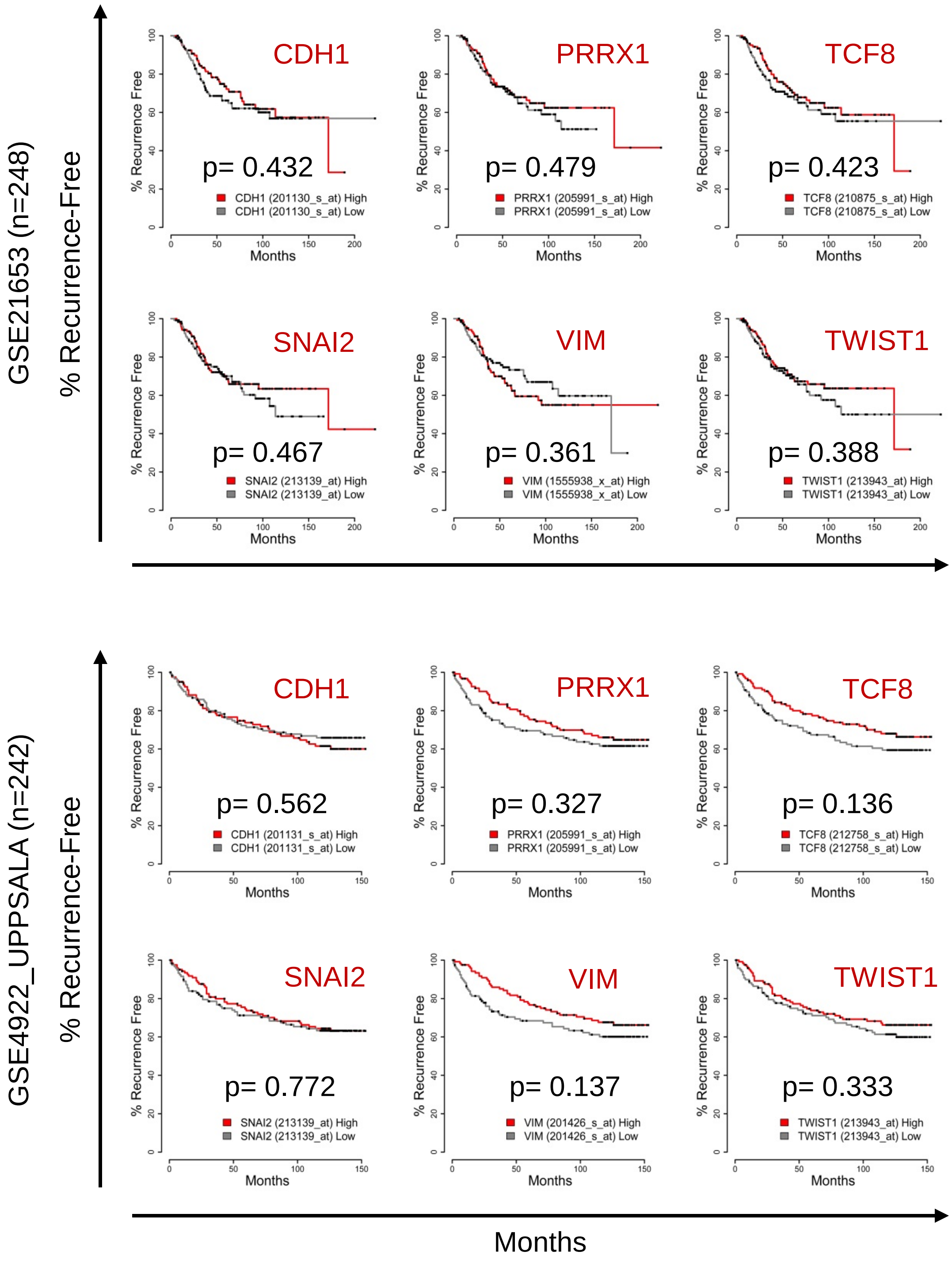

Suppl Figure. 7

Key EMT-related Factors, Survival Curves, Recurrence-Free Survival –Set 2

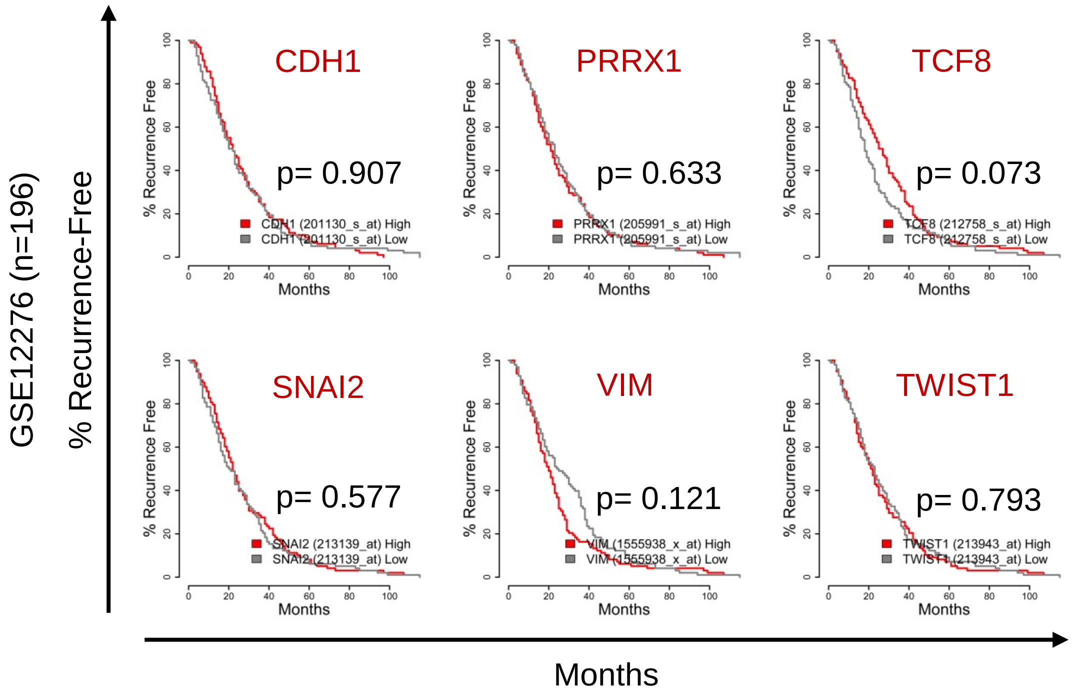

Suppl Figure. 8

Well-characterized EMT-/stem cell-related markers used for comparative analyses with the newly identified E6.5 gene expression signature

A.

Gene Name	Functional Role in EMT/Stem-cell Phenotype References (PMID#)
ACTA1	19909494, 22208948
ALDH1A1	23982874, 21280157
AR	24948871, 25307492
ATF2	17079470, 20067572
BMI1	25348805, 16778178
CD34	2469766, 11486732
CD44	12629218, 23046710
CDH1	18483246, 25079037
CDH2	23975425, 23975425
ERBB3	23937725, 23447672
ESR1	15223047, 25013076
ETS1	24556840, 22829018
FN1	23624917, 23912459
FOXC1	22645147, 23677979
FOXC2	23378344, 17537911
GATA3	20189993, 23354167
GSC	21392411, 20713713
KLF10	21980432
KLK3	22560078, 17587816
KRT18	19487819, 22439911
KRT5	18281472, 18301244
LEF1	21874052, 23613467
PECAM1	18796538, 15126319
PGR	20540763, 23640060
PROM1	21337465, 22469978
PRRX1	23201163, 24946010
SNAI1	23342249, 24638100
SNAI2	22385965, 23086238
SOX2	21822303, 24178749
SP1	17308095, 24906624
TCF3	23090119, 11445543
TCF4	22080605, 22232078
TCF8	23827675, 23412770
TGFB1	21555371, 24335925
THY1	25266422
TWIST1	15210113, 22266852
TWIST2	21602879, 23133563
VIM	17587825, 23412770
ZFHX1B	22594450, 20459606

B.

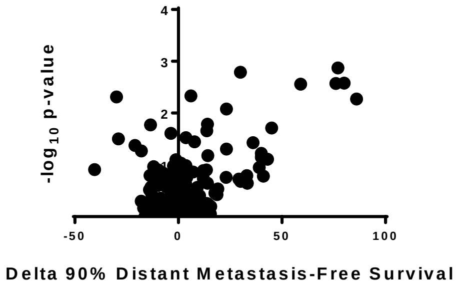

C.

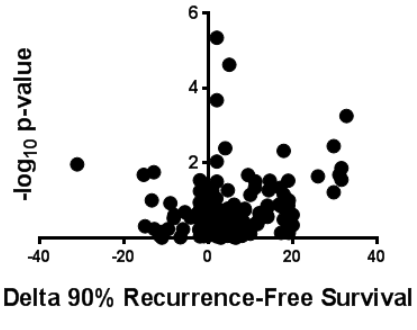

Suppl Figure. 9

Determining metastatic competence of commonly used prostate cancer cell lines based on concordance with the E6.5 gene expression signature.

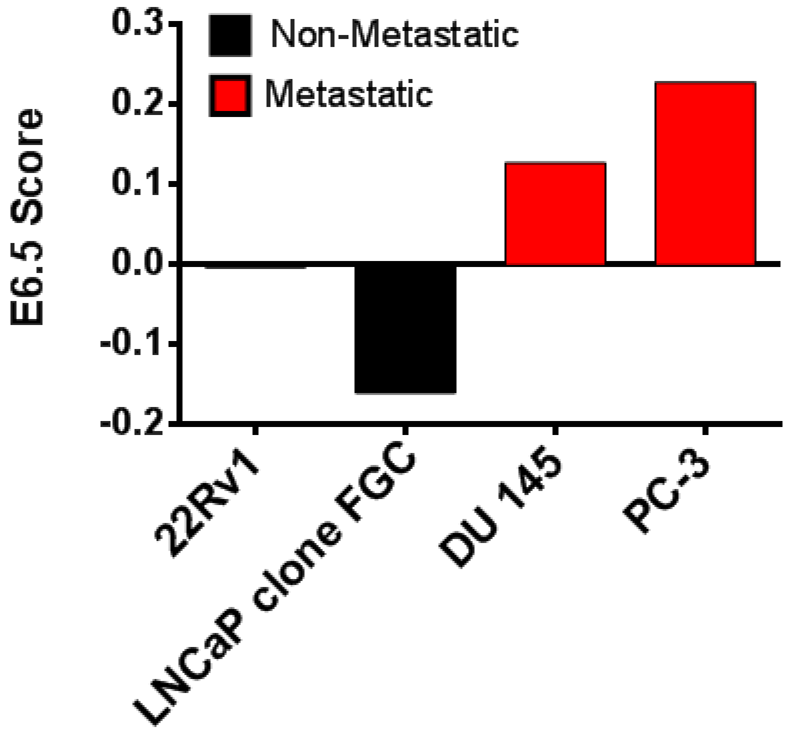

A novel embryonic plasticity gene signature that predicts metastatic competence and clinical outcome

Soundararajan *et. al.*

Supplementary Data

TABLES

Suppl Table-1: Complete list of genes constituting the E6.5-, and adult gene expression signatures. "Up" denotes significantly up-regulated genes and "Down" denotes significantly down-regulated genes.

Suppl Table-2: Functional gene networks operative in the newly identified E6.5 signature. The E6.5 embryonic gene expression signature was analyzed for significantly altered functional gene networks using Qiagen's Ingenuity Pathway Analysis. Shown are the notably up/down-regulated pathways operative in the identified signatures, and the identity of key genes that constitute the altered functional network.

FIGURES

Suppl Figure 1: Kaplan-Meier curves of distant metastasis-free survival (DMFS) in breast cancer patients stratified by expression of the E6.5-, the adult-, or the EMT gene expression signature, were plotted using patient data from four independent studies [1, 2, 3, 4] [Data from one of the cohorts is presented in Fig. 5, the remaining are presented here]. Log-rank tests were used to assess statistical significance of differences in DMFS based on concordance with each signature. $p<0.05$ was considered as the necessary cutoff for statistical significance.

Suppl Figure 2: Kaplan-Meier curves of recurrence-free survival (RFS) in breast cancer patients stratified by expression of the E6.5-, the adult-, or the EMT gene expression signature, were plotted using patient data from four independent studies [5, 6, 7, 8] [Data from one of the cohorts is presented in Fig. 5, the remaining are presented here]. Log-rank tests were used to assess statistical significance of differences in RFS based on concordance with each signature. $p<0.05$ was considered as the necessary cutoff for statistical significance.

Suppl Figure 3: A quantitative representation of the predicted relative metastatic abilities of various human breast cancer cell lines based on their E6.5- (**A**) or Adult- (**B**) scores.

Suppl Figures 4, 5: Kaplan-Meier curves of distant metastasis-free survival (DMFS) in breast cancer patients stratified by expression of CDH1, PRRX1, TCF-8, SNAI2, VIM and TWIST1, were plotted using patient data from three independent studies [GSE7390 [3], GSE11121 [4], GSE20685 [1]]. Log-rank tests were used to assess

statistical significance of differences in DMFS based on concordance with expression of each gene. p<0.05 was considered as the necessary cutoff for statistical significance.

Suppl Figures 6, 7: Kaplan-Meier curves of recurrence-free survival (RFS) in breast cancer patients stratified by expression of CDH1, PRRX1, TCF-8, SNAI2, VIM and TWIST1, were plotted using patient data from three independent studies [GSE12276 [6], GSE4922_UPPSALA [7], and GSE1653 [8]]. Log-rank tests were used to assess statistical significance of differences in RFS based on concordance with each signature. p<0.05 was considered as the necessary cutoff for statistical significance.

Suppl Figure 8: Well-characterized EMT- and stem cell-markers used for comparative analyses with the newly identified E6.5 gene expression signature. 39 genes were selected on the basis of their established EMT/stem cell-related functions. Panel **A** shows the full list of compared genes. The scatter plot in panel **B** shows compiled Kaplan-Meier curves of distant metastasis-free survival (DMFS) in breast cancer patients stratified by expression of EMT- and stem cell-markers listed in A, plotted using patient data from four independent studies [1, 2, 3, 4]. The scatter plot in panel **C** shows compiled Kaplan-Meier curves of recurrence-free survival (RFS) in breast cancer patients stratified by expression of EMT- and stem cell-markers listed in A, plotted using patient data from four independent studies [5, 6, 7, 8].

Suppl Figure 9: A quantitative representation of the predicted relative metastatic abilities of various prostate cancer cell lines based on their E6.5 scores.

Supplementary References:

1. **Kao KJ, Chang KM, Hsu HC, Huang AT. Correlation of microarray-based breast cancer molecular subtypes and clinical outcomes: implications for treatment optimization. *BMC Cancer* 11, 143 (2011).**

2. **Loi S*, et al.* Definition of clinically distinct molecular subtypes in estrogen receptor-positive breast carcinomas through genomic grade. *Journal of clinical oncology : official journal of the American Society of Clinical Oncology* 25, 1239-1246 (2007).**

3. **Desmedt C*, et al.* Strong time dependence of the 76-gene prognostic signature for node-negative breast cancer patients in the TRANSBIG multicenter independent validation series. *Clin Cancer Res* 13, 3207-3214 (2007).**

4. **Schmidt M*, et al.* The humoral immune system has a key prognostic impact in node-negative breast cancer. *Cancer Res* 68, 5405-5413 (2008).**

5. **Pawitan Y*, et al.* Gene expression profiling spares early breast cancer patients from adjuvant therapy: derived and validated in two population-based cohorts. *Breast Cancer Res* 7, R953-964 (2005).**

6. Bos PD*, et al.* Genes that mediate breast cancer metastasis to the brain. *Nature* 459, 1005-1009 (2009).

7. Ivshina AV*, et al.* Genetic reclassification of histologic grade delineates new clinical subtypes of breast cancer. *Cancer Res* 66, 10292-10301 (2006).

8. Sabatier R*, et al.* A gene expression signature identifies two prognostic subgroups of basal breast cancer. *Breast Cancer Res Treat* 126, 407-420 (2011).

Complete list of genes constituting the E6.5- and Adult gene expression signatures Suppl Table-1

Adult Up					
**Probe Set ID**	**Description**	**LocusLink Mouse**	**Gene Symbol Mouse**	**LocusLink Human**	**Gene Symbol Human**
gnf1m00181_a_at	acyl-Coenzyme A binding domain containing 5	74159	Acbd5	91452	ACBD5
gnf1m00184_a_at	carnitine palmitoyltransferase 1a, liver	12894	Cpt1a	1374	CPT1A
gnf1m00191_a_at	peroxiredoxin 5	54683	Prdx5	25824	PRDX5
gnf1m00192_a_at	matrix Gla protein	17313	Mgp	4256	MGP
gnf1m00197_s_at	transmembrane protein 59	56374	Tmem59	9528	TMEM59
gnf1m00229_a_at	testis-specific protein, Y-encoded-like 1	22110	Tspyl1	7259	TSPYL1
gnf1m00246_a_at	histone 1, H1c	50708	Hist1h1c	3006	HIST1H1C
gnf1m00260_a_at	receptor accessory protein 5	13476	Reep5	7905	REEP5
gnf1m00438_a_at	acylphosphatase 2, muscle type	75572	Acyp2	98	ACYP2
gnf1m00448_a_at	RIKEN cDNA 4930570C03 gene	67739	4930570C03Rik	55652	SLC48A1
gnf1m00471_a_at	tumor protein p53 inducible nuclear protein 2	68728	Trp53inp2	58476	TP53INP2
gnf1m00474_a_at	aldehyde dehydrogenase 1 family, member L1	107747	Aldh1l1	10840	ALDH1L1
gnf1m00523_a_at	signal transducer and activator of transcription 5B	20851	Stat5b	6777	STAT5B
gnf1m00529_a_at	leptin receptor overlapping transcript-like 1	68192	Leprotl1	23484	LEPROTL1
gnf1m00538_a_at	Rho guanine nucleotide exchange factor (GEF) 3	71704	Arhgef3	50650	ARHGEF3
gnf1m00644_a_at	cellular repressor of E1A-stimulated genes 1	433375	Creg1	8804	CREG1
gnf1m00651_a_at	osteopetrosis associated transmembrane protein 1	14628	Ostm1	28962	OSTM1
gnf1m00653_at	CDP-diacylglycerol synthase 1	74596	Cds1	1040	CDS1
gnf1m00697_at	sphingosine-1-phosphate phosphatase 1	81535	Sgpp1	81537	SGPP1
gnf1m00705_a_at	RIKEN cDNA 2310005E10 gene	67861	2310005E10Rik	57016	AKR1B10
gnf1m00706_a_at	CD74 antigen (invariant polypeptide of major histocompatibility complex, class II antigen-associated)	16149	Cd74	972	CD74
gnf1m00741_a_at	synaptosomal-associated protein	67474	Snap29	9342	SNAP29
gnf1m00781_a_at	a disintegrin and metallopeptidase domain 9 (meltrin gamma)	11502	Adam9	8754	ADAM9
gnf1m00800_a_at	aldehyde dehydrogenase family 3, subfamily A2				
gnf1m00820_a_at	baculoviral IAP repeat-containing 2	11797	Birc2	329	BIRC2
gnf1m00876_a_at	complement component 1, q subcomponent, alpha polypeptide	12259	C1qa	712	C1QA
gnf1m00878_a_at	complement component 1, q subcomponent, C chain	12262	C1qc	714	C1QC
gnf1m00904_a_at	chromobox homolog 4 (Drosophila Pc class)	12418	Cbx4	8535	CBX4
gnf1m00943_a_at	cold inducible RNA binding protein	12696	Cirbp	1153	CIRBP
gnf1m00946_a_at	circadian locomoter output cycles kaput	12753	Clock	9575	CLOCK
gnf1m01013_at	decorin	13179	Dcn	1634	DCN
gnf1m01054_a_at	endothelial differentiation sphingolipid G-protein-coupled receptor 1	13609	Edg1	1901	S1PR1
gnf1m01057_a_at	early growth response 1	13653	Egr1	1958	EGR1

Adult Up

gnf1m01115_a_at	ferrochelatase	14151	Fech	2235	FECH
gnf1m01143_a_at	insulin-like growth factor binding protein 7	29817	Igfbp7	3490	IGFBP7
gnf1m01146_a_at	Fyn proto-oncogene	14360	Fyn	2534	FYN
gnf1m01329_a_at	integral membrane protein 2B	16432	Itm2b	9445	ITM2B
gnf1m01581_a_at	cytochrome c oxidase, subunit VIIc	12867	Cox7c		
gnf1m01669_a_at	retinoblastoma 1	19645	Rb1	5925	RB1
gnf1m01952_a_at	ring finger protein 103	22644	Rnf103	7844	RNF103
gnf1m01966_a_at	zinc fingers and homeoboxes protein 1	22770	Zhx1	11244	ZHX1
gnf1m01974_s_at	ATP-binding cassette, sub-family G (WHITE), member 1	11307	Abcg1	9619	ABCG1
gnf1m02003_a_at	aldehyde dehydrogenase 2, mitochondrial	11669	Aldh2	217	ALDH2
gnf1m02056_a_at	complement component 1, q subcomponent, beta polypeptide	12260	C1qb	713	C1QB
gnf1m02123_s_at	complement component factor h	12628	Cfh	3075	CFH
gnf1m02128_a_at	creatine kinase, mitochondrial 1, ubiquitous	12716	Ckmt1	1159	CKMT1B
gnf1m02141_a_at	procollagen, type VI, alpha 1	12833	Col6a1	1291	COL6A1
gnf1m02231_a_at	CUG triplet repeat, RNA binding protein 2	14007	Cugbp2	10659	CELF2
gnf1m02312_a_at	amino-terminal enhancer of split	14797	Aes	166	AES
gnf1m02320_s_at	glutathione S-transferase, mu 1	14862	Gstm1	2949	GSTM5
gnf1m02323_a_at	glutathione transferase zeta 1 (maleylacetoacetate isomerase)	14874	Gstz1	2954	GSTZ1
gnf1m02334_s_at	histocompatibility 2, class II antigen A, alpha	14960	H2-Aa	3117	HLA-DQA1
gnf1m02335_s_at	histocompatibility 2, class II antigen E beta	14969	H2-Eb1	3123	HLA-DRB1
gnf1m02343_s_at	histocompatibility 2, T region locus 10	15024	H2-T10		
gnf1m02344_s_at	histocompatibility 2, T region locus 23	15040	H2-T23	3133	HLA-E
gnf1m02355_a_at	hexosaminidase B	15212	Hexb	3074	HEXB
gnf1m02405_a_at	interferon gamma receptor 1	15979	Ifngr1	3459	IFNGR1
gnf1m02491_a_at	phytanoyl-CoA hydroxylase	16922	Phyh	5264	PHYH
gnf1m02511_at	methyl-CpG binding domain protein 2	17191	Mbd2	8932	MBD2
gnf1m02557_a_at	nuclear receptor coactivator 1	17977	Ncoa1	8648	NCOA1
gnf1m02567_a_at	nuclear factor I/X	18032	Nfix	4784	NFIX
gnf1m02621_a_at	period homolog 2 (Drosophila)	18627	Per2	8864	PER2
gnf1m02653_a_at	phospholipid transfer protein	18830	Pltp	5360	PLTP
gnf1m02676_a_at	prion protein	19122	Prnp	5621	PRNP
gnf1m02799_a_at	sphingomyelin phosphodiesterase 1, acid lysosomal	20597	Smpd1	6609	SMPD1
gnf1m02835_a_at	syntaxin binding protein 3A	20912	Stxbp3a	6814	STXBP3
gnf1m02956_a_at	autocrine motility factor receptor	23802	Amfr	267	AMFR
gnf1m02984_a_at	monoglyceride lipase	23945	Mgll	11343	MGLL
gnf1m03020_a_at	ubiquitin-like 3	24109	Ubl3	5412	UBL3
gnf1m03120_a_at	clusterin	12759	Clu	1191	CLU
gnf1m03121_a_at	carboxypeptidase E	12876	Cpe	1363	CPE
gnf1m03167_s_at	lysozyme	17105	Lyzs		
gnf1m03171_a_at	methyl-CpG binding domain protein 1	17190	Mbd1	4152	MBD1

gnf1m03324_a_at	N-myc downstream regulated gene 2	29811	Ndrg2	57447	NDRG2
gnf1m03347_a_at	F-box and WD-40 domain protein 5	30839	Fbxw5	54461	FBXW5
gnf1m03361_a_at	acyl-Coenzyme A oxidase 1, palmitoyl	11430	Acox1	51	ACOX1
gnf1m03362_a_at	ATPase, class II, type 9A	11981	Atp9a	10079	ATP9A
gnf1m03368_a_at	ectonucleotide pyrophosphatase/phosphodiesterase 2	18606	Enpp2	5168	ENPP2
gnf1m03449_a_at	growth factor receptor bound protein 14	50915	Grb14	2888	GRB14
gnf1m03471_a_at	enoyl coenzyme A hydratase 1, peroxisomal	51798	Ech1	1891	ECH1
gnf1m03490_a_at	WW domain binding protein 2	22378	Wbp2	23558	WBP2
gnf1m03513_a_at	receptor (calcitonin) activity modifying protein 1	51801	Ramp1	10267	RAMP1
gnf1m03543_a_at	D site albumin promoter binding protein	13170	Dbp	1628	DBP
gnf1m03550_a_at	acyl-Coenzyme A dehydrogenase, very long chain	11370	Acadvl	37	ACADVL
gnf1m03555_a_at	thyrotroph embryonic factor	21685	Tef	7008	TEF
gnf1m03609_a_at	TBC1 domain family, member 8	54610	Tbc1d8	11138	TBC1D8
gnf1m03622_at	ATPase, H+ transporting, lysosomal accessory protein 1	54411	Atp6ap1	537	ATP6AP1
gnf1m03641_a_at	N-acylsphingosine amidohydrolase 2	54447	Asah2	56624	ASAH2
gnf1m03718_a_at	FXYD domain-containing ion transport regulator 1	56188	Fxyd1	5348	FXYD1
gnf1m03724_a_at	growth arrest specific 6	14456	Gas6	2621	GAS6
gnf1m03892_a_at	muscleblind-like 1 (Drosophila)	56758	Mbnl1	4154	MBNL1
gnf1m03955_a_at	aminolevulinic acid synthase 1	11655	Alas1	211	ALAS1
gnf1m03956_a_at	sphingomyelin phosphodiesterase, acid-like 3A	57319	Smpdl3a	10924	SMPDL3A
gnf1m04109_a_at	ribonuclease, RNase A family 4	58809	Rnase4	6038	RNASE4
gnf1m04146_a_at	C1GALT1-specific chaperone 1	59048	C1galt1c1	29071	C1GALT1C1
gnf1m04178_a_at	transformation related protein 53 inducible nuclear protein 1	60599	Trp53inp1	94241	TP53INP1
gnf1m04207_a_at	homocysteine-inducible, endoplasmic reticulum stress-inducible, ubiquitin-like domain member 1	64209	Herpud1	9709	HERPUD1
gnf1m04212_a_at	integral membrane protein 2C	64294	Itm2c	81618	ITM2C
gnf1m04241_s_at	low-density lipoprotein receptor-related protein 10	65107	Lrp10	26020	LRP10
gnf1m04336_a_at	f-box only protein 9	71538	Fbxo9	26268	FBXO9
gnf1m04356_a_at	thioredoxin interacting protein	56338	Txnip	10628	TXNIP
gnf1m04456_a_at	speckle-type POZ protein	20747	Spop	8405	SPOP
gnf1m04491_a_at	transmembrane emp24 domain containing 3	66111	Tmed3	23423	TMED3
gnf1m04503_a_at	interferon induced transmembrane protein 3	66141	Ifitm3	10410	IFITM3
gnf1m04552_a_at	RIKEN cDNA 1810037C20 gene	66294	1810037C20Rik	60343	FAM3A
gnf1m04572_a_at	dehydrogenase/reductase (SDR family) member 7	66375	Dhrs7	51635	DHRS7
gnf1m04575_a_at	NifU-like N-terminal domain containing	66383	Nifun	23479	ISCU
gnf1m04600_a_at	RIKEN cDNA 2810405K02 gene	66469	2810405K02Rik	127281	FAM213B

Adult Up

gnf1m04638_s_at	ORM1-like 3 (S. cerevisiae)	66612	Ormdl3	94103	ORMDL3
gnf1m04716_a_at	acyl-Coenzyme A dehydrogenase, short/branched chain	66885	Acadsb	36	ACADSB
gnf1m04733_a_at	scotin gene	66940	Scotin	51246	SHISA5
gnf1m04735_a_at	PQ loop repeat containing 1	66943	Pqlc1	80148	PQLC1
gnf1m04786_a_at	NADH dehydrogenase (ubiquinone) 1 alpha subcomplex, 6 (B14)	67130	Ndufa6	4700	NDUFA6
gnf1m04853_a_at	microtubule-associated protein 1 light chain 3 beta	67443	Map1lc3b	81631	MAP1LC3B
gnf1m05018_a_at	transmembrane BAX inhibitor motif containing 4	68212	Tmbim4	51643	TMBIM4
gnf1m05080_a_at	yippee-like 3 (Drosophila)	66090	Ypel3	83719	YPEL3
gnf1m05168_a_at	brain protein 44	70456	Brp44	25874	BRP44
gnf1m05171_a_at	RIKEN cDNA 5730469M10 gene	70564	5730469M10Rik	84293	C10orf58
gnf1m05226_at	RIKEN cDNA 1110007C02 gene	71784	1110007C02Rik		
gnf1m05237_a_at	RIKEN cDNA 2310001A20 gene	71881	2310001A20Rik	57136	C20orf3
gnf1m05357_a_at	neuronal pentraxin receptor	73340	Nptxr	23467	NPTXR
gnf1m05406_a_at	DNA-damage-inducible transcript 4	74747	Ddit4	54541	DDIT4
gnf1m05476_a_at	glutathione S-transferase kappa 1	76263	Gstk1	373156	GSTK1
gnf1m05545_a_at	cytoglobin	114886	Cygb	114757	CYGB
gnf1m05702_a_at	ectonucleotide pyrophosphatase/phosphodiesterase 5	83965	Enpp5	59084	ENPP5
gnf1m05864_a_at	tubby like protein 4	68842	Tulp4	56995	TULP4
gnf1m05894_a_at	growth hormone inducible transmembrane protein	66092	Ghitm	27069	GHITM
gnf1m05916_a_at	acyl-CoA synthetase short-chain family member 1	68738	Acss1	84532	ACSS1
gnf1m05959_a_at	oxidation resistance 1	170719	Oxr1	55074	OXR1
gnf1m06017_a_at	CD 81 antigen	12520	Cd81	975	CD81
gnf1m06051_a_at	WD repeat domain 23	28199	Wdr23	80344	DCAF11
gnf1m06054_a_at	SNF related kinase	20623	Snrk	54861	SNRK
gnf1m06058_a_at	insulin induced gene 2	72999	Insig2	51141	INSIG2
gnf1m06079_a_at	DNA segment, Chr 1, ERATO Doi 622, expressed	52392	D1Ertd622e	90355	C5orf30
gnf1m06244_a_at	Cd99 antigen-like 2	171486	Cd99l2	83692	CD99L2
gnf1m06322_a_at	HRAS like suppressor 3	225845	Hrasls3	11145	PLA2G16
gnf1m06323_s_at	HRAS like suppressor 3	225845	Hrasls3	11145	PLA2G16
gnf1m06378_s_at	trinucleotide repeat containing 6b	213988	Tnrc6b	23112	TNRC6B
gnf1m06460_a_at	Dip3 beta	216190	Dip3b	55198	APPL2
gnf1m06541_a_at	pleckstrin homology domain containing, family B (evectins) member 2	226971	Plekhb2	55041	PLEKHB2
gnf1m06564_a_at	hydroxyacyl-Coenzyme A dehydrogenase/3-ketoacyl-Coenzyme A thiolase/enoyl-Coenzyme A hydratase (trifunctional protein), beta subunit	231086	Hadhb	3032	HADHB
gnf1m06671_a_at	t-complex 11 (mouse) like 2	216198	Tcp11l2	255394	TCP11L2
gnf1m06711_a_at	tudor domain containing 7	100121	Tdrd7	23424	TDRD7

Adult Up

gnf1m07435_a_at	gamma-glutamyl hydrolase	14590	Ggh		
gnf1m07444_a_at	transforming growth factor, beta receptor II	21813	Tgfbr2	7048	TGFBR2
gnf1m07452_at	dickkopf homolog 3 (Xenopus laevis)	50781	Dkk3	27122	DKK3
gnf1m07462_a_at	N-glycanase 1	59007	Ngly1	55768	NGLY1
gnf1m07514_a_at	S100 calcium binding protein A16	67860	S100a16	140576	S100A16
gnf1m07625_a_at	CD47 antigen (Rh-related antigen, integrin-associated signal transducer)	16423	Cd47	961	CD47
gnf1m07635_a_at	endonuclease domain containing 1	71946	Endod1	23052	ENDOD1
gnf1m07759_a_at	chondroitin sulfate GalNAcT-2	78752	Galnact2	55454	CSGALNACT2
gnf1m07790_a_at	adaptor-related protein complex 1, sigma 2 subunit	108012	Ap1s2	8905	AP1S2
gnf1m07808_at	protein-L-isoaspartate (D-aspartate) O-methyltransferase domain containing 2	245867	Pcmtd2	55251	PCMTD2
gnf1m08011_at	nuclear transport factor 2-like export factor 2	237082	Nxt2	55916	NXT2
gnf1m08100_a_at	nuclear factor I/A	18027	Nfia	4774	NFIA
gnf1m08573_at	myotubularin related protein 6	219135	Mtmr6	9107	MTMR6
gnf1m08690_a_at	gephyrin	268566	Gphn	10243	GPHN
gnf1m08923_a_at	RIKEN cDNA 1500011K16 gene	67885	1500011K16Rik		
gnf1m09265_a_at	RIKEN cDNA 9130023F12 gene	216549	9130023F12Rik	54812	AFTPH
gnf1m09288_a_at	caveolin 2	12390	Cav2	858	CAV2
gnf1m09320_a_at	RIKEN cDNA 2210010L05 gene	98682	2210010L05Rik	54842	MFSD6
gnf1m09341_at	RIKEN cDNA 4933426M11 gene	217684	4933426M11Rik	9766	KIAA0247
gnf1m09439_a_at	SWI/SNF related, matrix associated, actin dependent regulator of chromatin, subfamily a, member 2	67155	Smarca2	6595	SMARCA2
gnf1m09477_a_at	hect domain and RLD 3	73998	Herc3	8916	HERC3
gnf1m09502_a_at	myosin XVIIIa	360013	Myo18a	399687	MYO18A
gnf1m09681_a_at	myosin regulatory light chain interacting protein	218203	Mylip	29116	MYLIP
gnf1m09695_a_at	hydroxyacyl glutathione hydrolase	14651	Hagh	3029	HAGH
gnf1m09698_s_at	RIKEN cDNA 4932438A13 gene	229227	4932438A13Rik	84162	KIAA1109
gnf1m09701_a_at	furry homolog (Drosophila)	320365	Fry	10129	FRY
gnf1m09784_a_at	T-cell leukemia translocation altered gene	102791	Tcta	6988	TCTA
gnf1m09827_a_at	cDNA sequence BC008155	214917	BC008155	65990	FAM173A
gnf1m09874_a_at	transferrin	22041	Trf	7018	TF
gnf1m09917_a_at	heat shock factor binding protein 1	68196	Hsbp1	3281	HSBP1
gnf1m09972_a_at	PTEN induced putative kinase 1	68943	Pink1	65018	PINK1
gnf1m10046_a_at	microtubule-associated protein 1 light chain 3 alpha	66734	Map1lc3a	84557	MAP1LC3A
gnf1m10103_a_at	DnaJ (Hsp40) homolog, subfamily B, member 4	67035	Dnajb4	11080	DNAJB4
gnf1m10151_a_at	proteasome (prosome, macropain) 28 subunit, beta	19188	Psme2	5721	PSME2
gnf1m10203_a_at	mal, T-cell differentiation protein 2	105853	Mal2	114569	MAL2
gnf1m10247_s_at	microtubule-associated protein 1 light chain 3 beta	67443	Map1lc3b	81631	MAP1LC3B

Adult Up

gnf1m10252_a_at	cytochrome b-561	13056	Cyb561	1534	CYB561
gnf1m10263_a_at	ilvB (bacterial acetolactate synthase)-like	216136	Ilvbl	10994	ILVBL
gnf1m10287_a_at	elongation factor RNA polymerase II 2	192657	Ell2	22936	ELL2
gnf1m10366_a_at	fibrinogen-like protein 2	14190	Fgl2	10875	FGL2
gnf1m10494_a_at	RIKEN cDNA 5033414D02 gene	67759	5033414D02Rik	55848	C9orf46
gnf1m10495_a_at	adenylate kinase 3	56248	Ak3	50808	AK3
gnf1m10525_at	lysozyme	17105	Lyzs		
gnf1m10549_a_at	Fc receptor, IgG, alpha chain transporter	14132	Fcgrt	2217	FCGRT
gnf1m10554_a_at	Mid1 interacting protein 1 (gastrulation specific G12-like (zebrafish))	68041	Mid1ip1	58526	MID1IP1
gnf1m10563_a_at	phenylalkylamine Ca2+ antagonist (emopamil) binding protein	13595	Ebp	10682	EBP
gnf1m10736_at	retinoblastoma-like 2	19651	Rbl2	5934	RBL2
gnf1m10842_a_at	RIKEN cDNA 1810009M01 gene	65963	1810009M01Rik	28959	TMEM176B
gnf1m10884_a_at	glutamate receptor, ionotropic, N-methyl D-asparate-associated protein 1 (glutamate binding)	66168	Grina	2907	GRINA
gnf1m10896_a_at	neural proliferation, differentiation and control gene 1	18146	Npdc1	56654	NPDC1
gnf1m10928_a_at	cystatin C	13010	Cst3	1471	CST3
gnf1m10941_a_at	acetyl-Coenzyme A dehydrogenase, long-chain	11363	Acadl	33	ACADL
gnf1m11009_a_at	plasma glutamate carboxypeptidase	54381	Pgcp	10404	PGCP
gnf1m11016_a_at	TSC22 domain family 3	14605	Tsc22d3	1831	TSC22D3
gnf1m11027_a_at	cysteine dioxygenase 1, cytosolic	12583	Cdo1	1036	CDO1
gnf1m11036_a_at	RIKEN cDNA 4632413K17 gene	216440	4632413K17Rik	10956	OS9
gnf1m11051_a_at	microsomal glutathione S-transferase 1	56615	Mgst1	4257	MGST1
gnf1m11055_a_at	RIKEN cDNA 6330583M11 gene	76192	6330583M11Rik	26090	ABHD12
gnf1m11130_s_at	mitogen activated protein binding protein interacting protein	83409	Mapbpip	28956	LAMTOR2
gnf1m11231_at	ubiquitin-conjugating enzyme E2B, RAD6 homology (S. cerevisiae)	22210	Ube2b	7320	UBE2B
gnf1m11254_a_at	DnaJ (Hsp40) homolog, subfamily B, member 10	56812	Dnajb10	3300	DNAJB2
gnf1m11299_a_at	beta-2 microglobulin	12010	B2m	567	B2M
gnf1m11306_a_at	ring finger protein 11	29864	Rnf11	26994	RNF11
gnf1m11314_a_at	very low density lipoprotein receptor	22359	Vldlr	7436	VLDLR
gnf1m11452_at	trans-golgi network protein	22134	Tgoln1		
gnf1m11540_a_at	abhydrolase domain containing 4	105501	Abhd4	63874	ABHD4
gnf1m11615_s_at	histocompatibility 2, class II antigen A, beta 1	14961	H2-Ab1		
gnf1m11666_a_at	Similar to mitogen-activated protein kinase kinase 5 homolog [Mus musculus]				
gnf1m11690_at	serine incorporator 3	26943	Serinc3	10955	SERINC3
gnf1m11695_at	vesicle-associated membrane protein 4	53330	Vamp4	8674	VAMP4
gnf1m11936_s_at	nuclear receptor subfamily 1, group D, member 2	353187	Nr1d2	9975	NR1D2

Adult Up

gnf1m11973_a_at	Sec11-like 3 (S. cerevisiae)	66286	Sec11l3	90701	SEC11C
gnf1m12007_a_at	NIMA (never in mitosis gene a)-related expressed kinase 7	59125	Nek7	140609	NEK7
gnf1m12129_a_at	sperm specific antigen 2	70599	Ssfa2	6744	SSFA2
gnf1m12188_a_at	B-cell leukemia/lymphoma 6	12053	Bcl6	604	BCL6
gnf1m12287_a_at	cyclin G2	12452	Ccng2	901	CCNG2
gnf1m12306_a_at	RAB3 GTPase activating protein subunit 1	226407	Rab3gap1	22930	RAB3GAP1
gnf1m12347_a_at	sterol-C5-desaturase (fungal ERG3, delta-5-desaturase) homolog (S. cerevisae)	235293	Sc5d	6309	SC5DL
gnf1m12546_a_at	tissue inhibitor of metalloproteinase 2	21858	Timp2	7077	TIMP2
gnf1m12552_a_at	nuclear factor I/B	18028	Nfib		
gnf1m12556_a_at	basic helix-loop-helix domain containing, class B2	20893	Bhlhb2	8553	BHLHE40
gnf1m12589_at	vesicle-associated membrane protein, associated protein B and C	56491	Vapb	9217	VAPB
gnf1m12701_a_at	scavenger receptor class B, member 2	12492	Scarb2	950	SCARB2
gnf1m12727_a_at	TMEM9 domain family, member B	56786	Tmem9b	56674	TMEM9B
gnf1m12741_a_at	progressive ankylosis	11732	Ank	56172	ANKH
gnf1m12778_a_at	preimplantation protein 4	74182	Prei4	56261	GPCPD1
gnf1m12807_a_at	tetraspanin 12	269831	Tspan12	23554	TSPAN12
gnf1m12871_a_at	phospholipase A2, group VII (platelet-activating factor acetylhydrolase, plasma)	27226	Pla2g7	7941	PLA2G7
gnf1m12981_a_at	vacuolar protein sorting 28 (yeast)	66914	Vps28	51160	VPS28
gnf1m12988_a_at	CD36 antigen	12491	Cd36	948	CD36
gnf1m13038_a_at	RAS p21 protein activator 3	19414	Rasa3	22821	RASA3
gnf1m13046_at	S-ADENOSYLHOMOCYSTEINE HYDROLASE-LIKE PROTEIN homolog [Homo sapiens]				
gnf1m13129_a_at	dynein light chain Tctex-type 3	67117	Dynlt3	6990	DYNLT3
gnf1m13132_a_at	chloride channel 4-2	12727	Clcn4-2	1183	CLCN4
gnf1m13156_a_at	transmembrane protein 66	67887	Tmem66	51669	TMEM66
gnf1m13227_a_at	caveolin, caveolae protein 1	12389	Cav1	857	CAV1
gnf1m13265_a_at	SPARC-like 1 (mast9, hevin)				
gnf1m13272_a_at	lysosomal membrane glycoprotein 2	16784	Lamp2	3920	LAMP2
gnf1m13285_a_at	lipin 1	14245	Lpin1	23175	LPIN1
gnf1m13398_s_at	RIKEN cDNA 3110001I20 gene	70354	3110001I20Rik	9728	SECISBP2L
gnf1m13506_a_at	RIKEN cDNA 6820401H01 gene	75743	6820401H01Rik		
gnf1m13556_at	RIKEN cDNA 5330406M23 gene	76671	5330406M23Rik		
gnf1m13784_a_at	TAP binding protein	21356	Tapbp	6892	TAPBP
gnf1m13804_a_at	Sel1 (suppressor of lin-12) 1 homolog (C. elegans)	20338	Sel1h	6400	SEL1L
gnf1m13829_at	RIKEN cDNA 3110073H01 gene	73201	3110073H01Rik		
gnf1m15276_a_at	sterol carrier protein 2, liver	20280	Scp2	6342	SCP2
gnf1m15367_a_at	avian musculoaponeurotic fibrosarcoma (v-maf) AS42 oncogene homolog	17132	Maf	4094	MAF

Adult Up

gnf1m15501_x_at	similar to H-2 class I histocompatibility antigen, L-D alpha chain precursor	547343	LOC547343		
gnf1m15626_a_at	homeodomain interacting protein kinase 1	15257	Hipk1	204851	HIPK1
gnf1m15635_a_at	cyclin G1	12450	Ccng1	900	CCNG1
gnf1m15720_x_at	Mouse clone IMAGE:3491638, mRNA, partial cds /cds=UNKNOWN /gb=BC003986 /gi=13278330 /len=2431 gnl|UG|Mm#S2006311				
gnf1m15731_x_at	hypothetical protein LOC626119	626119	LOC626119		
gnf1m15832_at	acyl-CoA synthetase long-chain family member 1	14081	Acsl1	2180	ACSL1
gnf1m15835_a_at	methyltransferase like 7A	70152	Mettl7a	25840	METTL7A
gnf1m15959_a_at	cDNA sequence BC005537	79555	BC005537	81688	C6orf62
gnf1m16027_at	microfibrillar-associated protein 3-like	71306	Mfap3l	9848	MFAP3L
gnf1m16207_at	DEP domain containing 6	97998	Depdc6	64798	DEPTOR
gnf1m16212_a_at	N-ethylmaleimide sensitive fusion protein attachment protein gamma	108123	Napg	8774	NAPG
gnf1m16251_at	hypothetical protein				
gnf1m16312_a_at	ELK4, member of ETS oncogene family	13714	Elk4	2005	ELK4
gnf1m16420_at	RIKEN cDNA C030044B11 gene	68128	C030044B11Rik		
gnf1m16422_at	transmembrane emp24 domain containing 8	382620	Tmed8	283578	TMED8
gnf1m16508_a_at	solute carrier family 36 (proton/amino acid symporter), member 1	215335	Slc36a1	206358	SLC36A1
gnf1m16543_a_at	Son of sevenless homolog 2 (Drosophila)	20663	Sos2	6655	SOS2
gnf1m16579_at	cryptochrome 2 (photolyase-like)	12953	Cry2	1408	CRY2
gnf1m16646_a_at	AXL receptor tyrosine kinase				
gnf1m16652_a_at	RIKEN cDNA 2010305C02 gene	380712	2010305C02Rik	727910	TLCD2
gnf1m16826_s_at	interferon, alpha-inducible protein 27	76933	Ifi27	83982	IFI27L2
gnf1m17366_s_at	serine/arginine-rich protein specific kinase 2	20817	Srpk2	6733	SRPK2
gnf1m17569_a_at	preproenkephalin 1	18619	Penk1	5179	PENK
gnf1m17891_at	FBJ osteosarcoma oncogene B				
gnf1m18318_a_at	DNA segment, Chr 12, ERATO Doi 647, expressed	52668	D12Ertd647e	3429	IFI27
gnf1m18361_at	bone morphogenic protein receptor, type II (serine/threonine kinase)	12168	Bmpr2	659	BMPR2
gnf1m18926_at	RIKEN cDNA A430107N12 gene	78432	A430107N12Rik		
gnf1m18977_a_at	proline-rich nuclear receptor coactivator 1	108767	Pnrc1	10957	PNRC1
gnf1m19003_at	epithelial membrane protein 2	13731	Emp2	2013	EMP2
gnf1m19004_a_at	RIKEN cDNA 5430417L22 gene	108676	5430417L22Rik		
gnf1m19563_at	unclassifiable				
gnf1m19829_a_at	transmembrane protein 64	100201	Tmem64	169200	TMEM64
gnf1m20802_at	ankyrin repeat domain 12	106585	Ankrd12	23253	ANKRD12
gnf1m21170_at	unclassifiable				
gnf1m21756_at	RIKEN cDNA 6330403M23 gene	109169	6330403M23Rik		
gnf1m21971_at	unclassifiable				

Adult Up

gnf1m22390_a_at	RIKEN cDNA D630014A15 gene	319666	D630014A15Rik		
gnf1m23003_s_at	DNA segment, Chr 11, Brigham & Women's Genetics 0434 expressed	52898	D11Bwg0434e	440400	RNASEK
gnf1m23004_a_at	RIKEN cDNA 2310043N10 gene	66961	2310043N10Rik		
gnf1m23009_at	RIKEN cDNA 2700089E24 gene	381820	2700089E24Rik		
gnf1m23071_at	similar to Retrovirus-related POL polyprotein (Endonuclease)	433762	LOC433762		
gnf1m23098_a_at	protein-L-isoaspartate (D-aspartate) O-methyltransferase 1				
gnf1m23157_a_at	succinate-Coenzyme A ligase, ADP-forming, beta subunit	20916	Sucla2	8803	SUCLA2
gnf1m23196_a_at	paraoxonase 2				
gnf1m23546_a_at	regulator of G-protein signaling 5	19737	Rgs5	8490	RGS5
gnf1m23606_s_at	SET domain containing (lysine methyltransferase) 7	73251	Setd7	80854	SETD7
gnf1m23696_a_at	ATP-binding cassette, sub-family A (ABC1), member 1	11303	Abca1	19	ABCA1
gnf1m24090_a_at	coronin, actin binding protein 2A	107684	Coro2a	7464	CORO2A
gnf1m25075_s_at	zinc finger protein 288				
gnf1m25187_at	cytoplasmic polyadenylation element binding protein 4	67579	Cpeb4	80315	CPEB4
gnf1m25374_a_at	leucyl/cystinyl aminopeptidase	240028	Lnpep	4012	LNPEP
gnf1m25749_at	hypothetical protein				
gnf1m25845_at	ERO1-like beta (S. cerevisiae)	67475	Ero1lb	56605	ERO1LB
gnf1m26335_at	ectonucleoside triphosphate diphosphohydrolase 5	12499	Entpd5	957	ENTPD5
gnf1m26442_s_at	trafficking protein particle complex 6B	78232	Trappc6b	122553	TRAPPC6B
gnf1m27110_a_at	RIKEN cDNA 5830471E12 gene	77208	5830471E12Rik		
gnf1m27182_a_at	A kinase (PRKA) anchor protein 13	75547	Akap13	11214	AKAP13
gnf1m27265_at	polyhomeotic-like 3 (Drosophila)	241915	Phc3	80012	PHC3
gnf1m27291_at	chloride channel 4-2		Clcn4-2		
gnf1m27309_s_at	sperm associated antigen 9	70834	Spag9	9043	SPAG9
gnf1m27877_at	hypothetical protein				
gnf1m27972_s_at	nuclear receptor coactivator 1				
gnf1m28386_at	RIKEN cDNA 1810015C04 gene	66270	1810015C04Rik	54463	FAM134B
gnf1m28549_at	vesicle-associated membrane protein 2	22318	Vamp2	6844	VAMP2
gnf1m28590_a_at	Ras and Rab interactor 2	74030	Rin2	54453	RIN2
gnf1m28683_a_at	CD44 antigen	12505	Cd44	960	CD44
gnf1m28737_a_at	3-oxoacid CoA transferase		Oxct		
gnf1m28740_s_at	CDP-diacylglycerol synthase 1	74596	Cds1	1040	CDS1
gnf1m28813_a_at	RIKEN cDNA 2310050N11 gene	66967	2310050N11Rik	80267	EDEM3
gnf1m28864_a_at	calcium binding protein 39	12283	Cab39	51719	CAB39
gnf1m28897_at	DNA segment, Chr 5, ERATO Doi 579, expressed	320661	D5Ertd579e	9778	KIAA0232
gnf1m28900_a_at	ethanolamine kinase 1	75320	Etnk1	55500	ETNK1

Adult Up

gnf1m28940_at	expressed sequence AI316807	102032	AI316807	114926	C8orf40
gnf1m29078_a_at	RIKEN cDNA 3110001I20 gene	70354	3110001I20Rik	9728	SECISBP2L
gnf1m29126_at	transmembrane protein 1	216131	Tmem1	7109	TRAPPC10
gnf1m29180_a_at	RIKEN cDNA 4933407C03 gene	74440	4933407C03Rik	80790	CMIP
gnf1m29291_a_at	RIKEN cDNA 0610007C21 gene	381629	0610007C21Rik	51374	C2orf28
gnf1m29444_at	RIKEN cDNA 4633402C03 gene RIKEN cDNA 4633402C03 gene mCG14896		4633402C03Rik		
gnf1m29555_at	RIKEN cDNA 1500004A08 gene	216505	1500004A08Rik	113791	PIK3IP1
gnf1m29607_a_at	protein phosphatase 3, catalytic subunit, alpha isoform				
gnf1m29661_a_at	protein phosphatase 1, regulatory (inhibitor) subunit 3C	53412	Ppp1r3c	5507	PPP1R3C
gnf1m29776_a_at	solute carrier family 41, member 1	98396	Slc41a1	254428	SLC41A1
gnf1m29808_a_at	transmembrane protein 106B	71900	Tmem106b	54664	TMEM106B
gnf1m29920_a_at	sestrin 1	140742	Sesn1	27244	SESN1
gnf1m29994_at	Protein FAM20B precursor				
gnf1m30002_at	ash1 (absent, small, or homeotic)-like (Drosophila)	192195	Ash1l	55870	ASH1L
gnf1m30015_a_at	Molecule interacting with Rab13 (MIRab13) (Fragment)				
gnf1m30024_a_at	calpastatin	12380	Cast	831	CAST
gnf1m30031_a_at	hypothetical protein				
gnf1m30100_a_at	cytoplasmic FMR1 interacting protein 2	76884	Cyfip2	26999	CYFIP2
gnf1m30171_a_at	poly (ADP-ribose) polymerase family, member 6	67287	Parp6	56965	PARP6
gnf1m30282_a_at	similar to CG1530-PA	432814	LOC432814		
gnf1m30315_a_at	thioredoxin domain containing 13	52837	Txndc13	56255	TMX4
gnf1m30419_a_at	inferred: forkhead box P1		Foxp1		
gnf1m30468_a_at	sortilin-related receptor, LDLR class A repeats-containing	20660	Sorl1	6653	SORL1
gnf1m30472_a_at	centaurin, beta 2	78618	Centb2	23527	ACAP2
gnf1m30539_a_at	thyroid hormone receptor associated protein 2	76199	Thrap2	23389	MED13L
gnf1m30540_a_at	ring finger protein 166	68718	Rnf166	115992	RNF166
gnf1m30579_at	glutaminase	14660	Gls	2744	GLS
gnf1m30607_a_at	RIKEN cDNA 2310047H23 gene	69672	2310047H23Rik	79770	TXNDC15
gnf1m30613_a_at	lemur tyrosine kinase 2	231876	Lmtk2	22853	LMTK2
gnf1m30655_a_at	testis expressed gene 2	21763	Tex2	55852	TEX2
gnf1m30750_a_at	chromobox homolog 6	494448	Cbx6	23466	CBX6
gnf1m30825_a_at	protein phosphatase 2, regulatory subunit B (B56), gamma isoform	26931	Ppp2r5c	5527	PPP2R5C
gnf1m30826_a_at	ectonucleotide pyrophosphatase/phosphodiesterase 4	224794	Enpp4	22875	ENPP4
gnf1m30856_at	transmembrane protein 23	208449	Tmem23	259230	SGMS1
gnf1m30872_a_at	transmembrane 7 superfamily member 3				
gnf1m30900_a_at	mcf.2 transforming sequence-like	17207	Mcf2l	23263	MCF2L

Adult Up

gnf1m30985_a_at	phosphatidylinositol 3-kinase, regulatory subunit, polypeptide 1 (p85 alpha)	18708	Pik3r1	5295	PIK3R1
gnf1m31028_at	actin-binding LIM protein 1	226251	Ablim1	3983	ABLIM1
gnf1m31048_a_at	ENSMUST00000039876 transcript (in rel.37.34e)				
gnf1m31065_a_at	myeloid/lymphoid or mixed-lineage leukemia 3	231051	Mll3	58508	MLL3
gnf1m31071_at	TBC1 domain family, member 4	210789	Tbc1d4	9882	TBC1D4
gnf1m31117_a_at	transmembrane and coiled coil domains 3	319880	Tmcc3	57458	TMCC3
gnf1m31122_a_at	cDNA sequence BC033915	70661	BC033915	23387	SIK3
gnf1m31194_a_at	interferon regulatory factor 2 binding protein 2	270110	Irf2bp2	359948	IRF2BP2
gnf1m32179_x_at	histocompatibility 2, D region locus 1	14964	H2-D1		
gnf1m32767_at	hypothetical protein				
gnf1m32879_x_at	obsolete Celera prediction mCT134077				
gnf1m35284_at	uncharacterized Celera prediction mCG1040029				

Adult Down					
**Probe Set ID**	**Description**	**LocusLink Mouse**	**Gene Symbol Mouse**	**LocusLink Human**	**Gene Symbol Human**
gnf1m00015_a_at	PWP2 (periodic tryptophan protein) homolog, yeast	110816	Pwp2h	5822	PWP2
gnf1m00027_at	ribosomal protein L4	67891	Rpl4	6124	RPL4
gnf1m00087_a_at	DNA segment, Chr 15, ERATO Doi 785, expressed	52683	D15Ertd785e	29781	NCAPH2
gnf1m00122_a_at	cullin 7	66515	Cul7	9820	CUL7
gnf1m00145_a_at	v-myc myelocytomatosis viral related oncogene, neuroblastoma derived (avian)	18109	Mycn	4613	MYCN
gnf1m00146_at	karyopherin (importin) beta 1	16211	Kpnb1	3837	KPNB1
gnf1m00148_a_at	asp (abnormal spindle)-like, microcephaly associated (Drosophila)	12316	Aspm	259266	ASPM
gnf1m00153_a_at	golgi phosphoprotein 2	105348	Golph2	51280	GOLM1
gnf1m00178_s_at	nucleoporin 43	69912	Nup43	348995	NUP43
gnf1m00195_s_at	thymidylate synthase	22171	Tyms	7298	TYMS
gnf1m00282_a_at	budding uninhibited by benzimidazoles 1 homolog (S. cerevisiae)	12235	Bub1	699	BUB1
gnf1m00294_at	TSR1, 20S rRNA accumulation, homolog (yeast)	104662	Tsr1	55720	TSR1
gnf1m00358_s_at	minichromosome maintenance deficient 10 (S. cerevisiae)	70024	Mcm10	55388	MCM10
gnf1m00418_s_at	RIKEN cDNA 9430010O03 gene	234023	9430010O03Rik	55082	ARGLU1
gnf1m00426_a_at	RIKEN cDNA 1700021I09 gene	67619	1700021I09Rik	28987	NOB1
gnf1m00435_a_at	ribosomal L1 domain containing 1	66409	Rsl1d1	26156	RSL1D1
gnf1m00442_a_at	cell division cycle associated 4	71963	Cdca4	55038	CDCA4
gnf1m00449_a_at	carnitine deficiency-associated gene expressed in ventricle 3	321022	Cdv3	55573	CDV3
gnf1m00476_a_at	HLA-B-associated transcript 1A	53817	Bat1a	7919	DDX39B
gnf1m00499_a_at	excision repair cross-complementing rodent repair deficiency complementation group 6 - like	236930	Ercc6l	54821	ERCC6L
gnf1m00511_a_at	protein arginine N-methyltransferase 3	71974	Prmt3	10196	PRMT3
gnf1m00528_a_at	RIKEN cDNA 3300001M20 gene	66926	3300001M20Rik		
gnf1m00559_a_at	solute carrier family 2 (facilitated glucose transporter), member 3	20527	Slc2a3	6515	SLC2A3
gnf1m00563_a_at	polo-like kinase 4 (Drosophila)	20873	Plk4	10733	PLK4
gnf1m00567_a_at	polycomb group ring finger 6	71041	Pcgf6	84108	PCGF6
gnf1m00642_at	nuclear receptor subfamily 6, group A, member 1	14536	Nr6a1	2649	NR6A1
gnf1m00701_a_at	nucleoporin 133	234865	Nup133	55746	NUP133

Adult Down

gnf1m00730_a_at	baculoviral IAP repeat-containing 5	11799	Birc5	332	BIRC5
gnf1m00774_a_at	actin, beta, cytoplasmic	11461	Actb	60	ACTB
gnf1m00776_a_at	activin receptor IIB	11481	Acvr2b	93	ACVR2B
gnf1m00785_a_at	poly (ADP-ribose) polymerase family, member 1	11545	Parp1	142	PARP1
gnf1m00788_a_at	alpha fetoprotein	11576	Afp	174	AFP
gnf1m00855_a_at	prohibitin 2	12034	Phb2	11331	PHB2
gnf1m00877_a_at	complement component 1, q subcomponent binding protein	12261	C1qbp	708	C1QBP
gnf1m00906_a_at	cyclin B2	12442	Ccnb2	9133	CCNB2
gnf1m00927_a_at	centromere autoantigen A	12615	Cenpa	1058	CENPA
gnf1m01053_a_at	ect2 oncogene	13605	Ect2	1894	ECT2
gnf1m01086_a_at	enhancer of rudimentary homolog (Drosophila)	13877	Erh	2079	ERH
gnf1m01098_a_at	enhancer of zeste homolog 2 (Drosophila)	14056	Ezh2	2146	EZH2
gnf1m01109_a_at	fibrillarin	14113	Fbl	2091	FBL
gnf1m01237_s_at	histone deacetylase 1	433759	Hdac1	3065	HDAC1
gnf1m01240_a_at	helicase, lymphoid specific	15201	Hells	3070	HELLS
gnf1m01321_a_at	Iroquois related homeobox 3 (Drosophila)	16373	Irx3	79191	IRX3
gnf1m01348_a_at	kinesin family member 4	16571	Kif4	24137	KIF4A
gnf1m01419_a_at	minichromosome maintenance deficient 2 mitotin (S. cerevisiae)	17216	Mcm2	4171	MCM2
gnf1m01420_s_at	minichromosome maintenance deficient 7 (S. cerevisiae)	17220	Mcm7	4176	MCM7
gnf1m01456_a_at	nudix (nucleoside diphosphate linked moiety X)-type motif 1	17766	Nudt1	4521	NUDT1
gnf1m01495_a_at	nidogen 2	18074	Nid2	22795	NID2
gnf1m01513_x_at	nucleophosmin 1	18148	Npm1	4869	NPM1
gnf1m01617_a_at	DNA primase, p49 subunit	19075	Prim1	5557	PRIM1
gnf1m01682_a_at	brain expressed gene 1	19716	Bex1	55859	BEX1
gnf1m01688_a_at	ribonucleic acid binding protein S1	19826	Rnps1	10921	RNPS1
gnf1m01697_a_at	RNA polymerase 1-1	20016	Rpo1-1	9533	POLR1C
gnf1m01709_a_at	ribonucleotide reductase M1	20133	Rrm1	6240	RRM1
gnf1m01710_a_at	ribonucleotide reductase M2	20135	Rrm2	6241	RRM2
gnf1m01750_a_at	seven in absentia 1B	20438	Siah1b		
gnf1m01863_a_at	thymidine kinase 1	21877	Tk1	7083	TK1
gnf1m01864_s_at	thymidine kinase 1	21877	Tk1	7083	TK1
gnf1m01898_a_at	Ttk protein kinase	22137	Ttk	7272	TTK
gnf1m01949_a_at	yes-associated protein 1	22601	Yap1	10413	YAP1
gnf1m02019_a_at	apurinic/apyrimidinic endonuclease 1	11792	Apex1	328	APEX1

Adult Down

gnf1m02048_a_at	nerve growth factor receptor (TNFRSF16) associated protein 1	12070	Ngfrap1	27018	NGFRAP1
gnf1m02054_a_at	budding uninhibited by benzimidazoles 1 homolog, beta (S. cerevisiae)	12236	Bub1b	701	BUB1B
gnf1m02087_a_at	serine (or cysteine) peptidase inhibitor, clade H, member 1	12406	Serpinh1	871	SERPINH1
gnf1m02089_a_at	cyclin A2	12428	Ccna2	890	CCNA2
gnf1m02091_a_at	chaperonin subunit 3 (gamma)	12462	Cct3	7203	CCT3
gnf1m02092_s_at	chaperonin subunit 3 (gamma)	12462	Cct3	7203	CCT3
gnf1m02094_a_at	chaperonin subunit 6a (zeta)	12466	Cct6a	908	CCT6A
gnf1m02274_a_at	phosphoribosylglycinamide formyltransferase	14450	Gart	2618	GART
gnf1m02363_a_at	H2A histone family, member X	15270	H2afx	3014	H2AFX
gnf1m02373_x_at	heterogeneous nuclear ribonucleoprotein A1	15382	Hnrpa1	3178	HNRNPA1
gnf1m02406_a_at	insulin-like growth factor 2	16002	Igf2	3481	IGF2
gnf1m02407_a_at	insulin-like growth factor 2 receptor	16004	Igf2r	3482	IGF2R
gnf1m02479_x_at	lactate dehydrogenase A	16828	Ldha	3939	LDHA
gnf1m02485_a_at	ligase I, DNA, ATP-dependent	16881	Lig1	3978	LIG1
gnf1m02488_a_at	lamin B1	16906	Lmnb1	4001	LMNB1
gnf1m02517_a_at	midkine	17242	Mdk	4192	MDK
gnf1m02526_s_at	MARCKS-like 1	17357	Marcksl1	65108	MARCKSL1
gnf1m02537_a_at	mutS homolog 6 (E. coli)	17688	Msh6	2956	MSH6
gnf1m02582_a_at	ubiquitin-like, containing PHD and RING finger domains, 1	18140	Uhrf1	29128	UHRF1
gnf1m02610_a_at	proliferating cell nuclear antigen	18538	Pcna	5111	PCNA
gnf1m02651_a_at	polo-like kinase 1 (Drosophila)	18817	Plk1	5347	PLK1
gnf1m02657_a_at	polymerase (DNA directed), delta 1, catalytic subunit	18971	Pold1	5424	POLD1
gnf1m02658_a_at	polymerase (DNA directed), epsilon	18973	Pole	5426	POLE
gnf1m02707_a_at	RAN binding protein 1	19385	Ranbp1	5902	RANBP1
gnf1m02735_at	RuvB-like protein 2	20174	Ruvbl2	10856	RUVBL2
gnf1m02770_a_at	Shc SH2-domain binding protein 1	20419	Shcbp1	79801	SHCBP1
gnf1m02785_a_at	solute carrier family 2 (facilitated glucose transporter), member 1	20525	Slc2a1	6513	SLC2A1
gnf1m02798_a_at	survival motor neuron 1	20595	Smn1	6606	SMN1
gnf1m02865_a_at	tripartite motif protein 28	21849	Trim28	10155	TRIM28
gnf1m02866_a_at	timeless homolog (Drosophila)	21853	Timeless	8914	TIMELESS
gnf1m02886_a_at	topoisomerase (DNA) II alpha	21973	Top2a	7153	TOP2A
gnf1m02915_a_at	valyl-tRNA synthetase 2	22321	Vars2	7407	VARS
gnf1m02960_a_at	carbonic anhydrase 14	23831	Car14	23632	CA14
gnf1m02961_a_at	craniofacial development protein 1	23837	Cfdp1	10428	CFDP1
gnf1m03074_a_at	Rac GTPase-activating protein 1				

Adult Down

gnf1m03141_a_at	EMG1 nucleolar protein homolog (S. cerevisiae)	14791	Emg1	10436	EMG1
gnf1m03148_a_at	hyaluronan mediated motility receptor (RHAMM)	15366	Hmmr	3161	HMMR
gnf1m03153_a_at	heat shock protein 110	15505	Hsp110	10808	HSPH1
gnf1m03185_x_at	ornithine decarboxylase, structural 1	18263	Odc1	4953	ODC1
gnf1m03253_a_at	Ras-GTPase-activating protein SH3-domain binding protein	27041	RP23-336J1.4	10146	G3BP1
gnf1m03258_a_at	DBF4 homolog (S. cerevisiae)	27214	Dbf4	10926	DBF4
gnf1m03339_a_at	translocase of inner mitochondrial membrane 8 homolog a (yeast)	30058	Timm8a	1678	TIMM8A
gnf1m03356_s_at	Cd27 binding protein (Hindu God of destruction)	30954	Siva	10572	SIVA1
gnf1m03373_a_at	hormonally upregulated Neu-associated kinase	26559	Hunk	30811	HUNK
gnf1m03378_a_at	heat shock protein 14	50497	Hspa14	51182	HSPA14
gnf1m03413_a_at	high mobility group AT-hook 1	15361	Hmga1	3159	HMGA1
gnf1m03414_a_at	S-adenosylhomocysteine hydrolase	269378	Ahcy	191	AHCY
gnf1m03429_a_at	inner centromere protein	16319	Incenp	3619	INCENP
gnf1m03433_a_at	glypican 3	14734	Gpc3	2719	GPC3
gnf1m03458_a_at	cytidine 5'-triphosphate synthase	51797	Ctps	1503	CTPS
gnf1m03466_a_at	peroxiredoxin 4	53381	Prdx4	10549	PRDX4
gnf1m03474_a_at	nuclear autoantigenic sperm protein (histone-binding)	50927	Nasp	4678	NASP
gnf1m03518_s_at	CDC28 protein kinase 1b	54124	Cks1b	1163	CKS1B
gnf1m03558_a_at	septin 9	53860	'Sept9	10801	40429
gnf1m03725_a_at	Down syndrome critical region homolog 2 (human)	56088	Dscr2	8624	PSMG1
gnf1m03733_a_at	DEAD (Asp-Glu-Ala-Asp) box polypeptide 21	56200	Ddx21	9188	DDX21
gnf1m03756_a_at	teratocarcinoma expressed, serine rich	56306	Tera		
gnf1m03766_a_at	actin-like 6A	56456	Actl6a	86	ACTL6A
gnf1m03775_a_at	RuvB-like protein 1	56505	Ruvbl1	8607	RUVBL1
gnf1m03787_a_at	origin recognition complex, subunit 6-like (S. cerevisiae)	56452	Orc6l	23594	ORC6
gnf1m03797_a_at	ubiquitin-like 1 (sentrin) activating enzyme E1A	56459	Uble1a	10055	SAE1
gnf1m03961_a_at	geminin	57441	Gmnn	51053	GMNN
gnf1m04036_s_at	RIKEN cDNA 2400006H24 gene	57785	2400006H24Rik		
gnf1m04128_a_at	nucleoporin 160	59015	Nup160	23279	NUP160
gnf1m04137_a_at	protein arginine N-methyltransferase 4	59035	Prmt4	10498	CARM1
gnf1m04262_x_at	enolase 1, alpha non-neuron	13806	Eno1	2023	ENO1

Adult Down

gnf1m04264_a_at	H19 fetal liver mRNA	14955	H19		
gnf1m04286_a_at	RIKEN cDNA 2410015N17 gene	66422	2410015N17Rik	79077	DCTPP1
gnf1m04287_a_at	PDZ binding kinase	52033	Pbk	55872	PBK
gnf1m04299_s_at	junction adhesion molecule 3	83964	Jam3	83700	JAM3
gnf1m04301_a_at	cell division cycle associated 1	66977	Cdca1	83540	NUF2
gnf1m04316_a_at	phosphoglycerate mutase 1	18648	Pgam1	5223	PGAM1
gnf1m04333_a_at	chromosome segregation 1-like (S. cerevisiae)	110750	Cse1l	1434	CSE1L
gnf1m04453_a_at	Ly1 antibody reactive clone	17089	Lyar	55646	LYAR
gnf1m04468_a_at	FtsJ homolog 3 (E. coli)	56095	Ftsj3	117246	FTSJ3
gnf1m04509_a_at	nuclear import 7 homolog (S. cerevisiae)	66164	Nip7	51388	NIP7
gnf1m04521_x_at	CDC28 protein kinase regulatory subunit 2	66197	Cks2	1164	CKS2
gnf1m04595_a_at	splicing factor, arginine/serine rich 9	108014	Sfrs9	8683	SRSF9
gnf1m04604_a_at	RIKEN cDNA 2010309E21 gene	66488	2010309E21Rik		
gnf1m04613_a_at	RWD domain containing 1	66521	Rwdd1	51389	RWDD1
gnf1m04628_a_at	WD repeat domain 57 (U5 snRNP specific)	66585	Wdr57	9410	SNRNP40
gnf1m04715_s_at	basic leucine zipper and W2 domains 1	66882	Bzw1		
gnf1m04737_a_at	cell division cycle associated 7	66953	Cdca7		
gnf1m04738_s_at	cell division cycle associated 7	66953	Cdca7		
gnf1m04765_s_at	arginyl-tRNA synthetase	104458	Rars	5917	RARS
gnf1m04781_a_at	ribosomal protein L14	67115	Rpl14	9045	RPL14
gnf1m04802_a_at	ubiquitin-conjugating enzyme E2T (putative)	67196	Ube2t	29089	UBE2T
gnf1m04804_a_at	RIKEN cDNA 2810037C14 gene	67211	2810037C14Rik	83787	ARMC10
gnf1m04809_s_at	gem (nuclear organelle) associated protein 6	67242	Gemin6	79833	GEMIN6
gnf1m04873_a_at	methyltransferase 10 domain containing	67493	Mett10d	79066	METTL16
gnf1m04937_a_at	peptidylprolyl isomerase D (cyclophilin D)	67738	Ppid	5481	PPID
gnf1m04951_a_at	RIKEN cDNA 2610027L16 gene	67842	2610027L16Rik	161424	C14orf21
gnf1m04954_a_at	cell division cycle associated 5	67849	Cdca5	113130	CDCA5
gnf1m04968_a_at	pyrophosphatase (inorganic) 1	67895	Ppa1	5464	PPA1
gnf1m05021_a_at	nudix (nucleoside diphosphate linked moiety X)-type motif 21	68219	Nudt21	11051	NUDT21
gnf1m05027_a_at	nucleolar protein family A, member 2	52530	Nola2	55651	NHP2
gnf1m05058_a_at	PHD finger protein 5A	68479	Phf5a	84844	PHF5A
gnf1m05062_a_at	ubiquitin-conjugating enzyme E2C	68612	Ube2c	11065	UBE2C
gnf1m05266_a_at	RIKEN cDNA 2610003J06 gene	72106	2610003J06Rik	339123	JMJD8
gnf1m05281_a_at	RIKEN cDNA 2610028A01 gene	72400	2610028A01Rik	54984	PINX1
gnf1m05297_a_at	nucleoporin 37	69736	Nup37	79023	NUP37
gnf1m05566_a_at	LSM2 homolog, U6 small nuclear RNA associated (S. cerevisiae)	27756	Lsm2	57819	LSM2
gnf1m05599_a_at	uridine-cytidine kinase 2	80914	Uck2	7371	UCK2
gnf1m05719_a_at	threonyl-tRNA synthetase	110960	Tars	6897	TARS

Adult Down

gnf1m05768_s_at	suppressor of Ty 16 homolog (S. cerevisiae)	114741	Supt16h	11198	SUPT16H
gnf1m05778_at	nucleoporin 62	18226	Nup62	23636	NUP62
gnf1m06076_a_at	lamin B receptor	98386	Lbr	3930	LBR
gnf1m06120_a_at	PWP1 homolog (S. cerevisiae)	103136	Pwp1	11137	PWP1
gnf1m06160_a_at	WD repeat domain 74	107071	Wdr74	54663	WDR74
gnf1m06288_a_at	methylenetetrahydrofolate dehydrogenase (NADP+ dependent), methenyltetrahydrofolate cyclohydrolase, formyltetrahydrofolate synthase	108156	Mthfd1	4522	MTHFD1
gnf1m06331_a_at	kinesin family member 18A	228421	Kif18a	81930	KIF18A
gnf1m06372_s_at	heterogeneous nuclear ribonucleoprotein L-like	72692	Hnrpll	92906	HNRPLL
gnf1m06384_a_at	barren homolog (Drosophila)	215387	Brrn1	23397	NCAPH
gnf1m06409_a_at	exosome component 2	227715	Exosc2	23404	EXOSC2
gnf1m06415_a_at	misato homolog 1 (Drosophila)	229524	Msto1	55154	MSTO1
gnf1m06580_a_at	THUMP domain containing 1	233802	Thumpd1	55623	THUMPD1
gnf1m06602_a_at	polymerase (RNA) II (DNA directed) polypeptide H	245841	Polr2h	5437	POLR2H
gnf1m06619_at	lin-28 homolog (C. elegans)	83557	Lin28	79727	LIN28A
gnf1m06649_a_at	cDNA sequence BC025462	208836	BC025462	55215	FANCI
gnf1m06695_a_at	splicing factor, arginine/serine-rich 7	225027	Sfrs7	6432	SRSF7
gnf1m06721_a_at	RIKEN cDNA 2810406C15 gene	68298	2810406C15Rik	9918	NCAPD2
gnf1m07338_a_at	achalasia, adrenocortical insufficiency, alacrimia	223921	Aaas	8086	AAAS
gnf1m07342_a_at	glutamate-rich WD repeat containing 1	101612	Grwd1	83743	GRWD1
gnf1m07367_a_at	guanine nucleotide binding protein-like 3 (nucleolar)	30877	Gnl3	26354	GNL3
gnf1m07433_a_at	flap structure specific endonuclease 1	14156	Fen1	2237	FEN1
gnf1m07541_a_at	JTV1 gene	231872	Jtv1	7965	AIMP2
gnf1m07632_a_at	nucleoporin 93	71805	Nup93	9688	NUP93
gnf1m07642_a_at	RIKEN cDNA 2610510J17 gene	72155	2610510J17Rik	55839	CENPN
gnf1m07740_a_at	CDK2 (cyclin-dependent kinase 2)-associated protein 1	13445	Cdk2ap1	8099	CDK2AP1
gnf1m07787_a_at	DNA segment, Chr 2, Wayne State University 81, expressed	227695	D2Wsu81e	51490	C9orf114
gnf1m08113_at	melanoma associated antigen (mutated) 1-like 1	245631	Mum1l1	139221	MUM1L1
gnf1m08188_a_at	methylenetetrahydrofolate dehydrogenase (NADP+ dependent) 1-like	270685	Mthfd1l	25902	MTHFD1L
gnf1m08318_a_at	HIRA interacting protein 3	233876	Hirip3	8479	HIRIP3
gnf1m08776_a_at	RIKEN cDNA E130012A19 gene	103551	E130012A19Rik	100170841	C17orf96

Adult Down

gnf1m08853_a_at	nucleosome assembly protein 1-like 1	53605	Nap1l1	4673	NAP1L1
gnf1m08856_at	splicing factor, arginine/serine-rich 3 (SRp20)	20383	Sfrs3	6428	SRSF3
gnf1m08857_a_at	splicing factor, arginine/serine-rich 3 (SRp20)	20383	Sfrs3	6428	SRSF3
gnf1m08913_a_at	histone aminotransferase 1	107435	Hat1	8520	HAT1
gnf1m08927_at	TGFB-induced factor 2	228839	Tgif2	60436	TGIF2
gnf1m09211_s_at	RAN, member RAS oncogene family	19384	Ran	5901	RAN
gnf1m09242_a_at	essential meiotic endonuclease 1 homolog 1 (S. pombe)	268465	Eme1	146956	EME1
gnf1m09302_a_at	sal-like 4 (Drosophila)	99377	Sall4	57167	SALL4
gnf1m09335_a_at	TAF9 RNA polymerase II, TATA box binding protein (TBP)-associated factor	108143	Taf9	6880	TAF9
gnf1m09354_s_at	stem-loop binding protein	20492	Slbp	7884	SLBP
gnf1m09360_a_at	heat shock protein 1 (chaperonin)	15510	Hspd1	3329	HSPD1
gnf1m09384_a_at	UTP20, small subunit (SSU) processome component, homolog (yeast)	70683	Utp20	27340	UTP20
gnf1m09394_a_at	HEAT repeat containing 1	217995	Heatr1	55127	HEATR1
gnf1m09414_a_at	SMC4 structural maintenance of chromosomes 4-like 1 (yeast)	70099	Smc4l1	10051	SMC4
gnf1m09443_a_at	expressed sequence C79407	217653	C79407	55320	MIS18BP1
gnf1m09452_s_at	cyclin D3	12445	Ccnd3	896	CCND3
gnf1m09470_x_at	3-phosphoglycerate dehydrogenase	236539	Phgdh	26227	PHGDH
gnf1m09489_a_at	RIKEN cDNA 2810025M15 gene	69953	2810025M15Rik		
gnf1m09509_a_at	WD repeat domain 55	67936	Wdr55	54853	WDR55
gnf1m09557_a_at	polymerase (RNA) II (DNA directed) polypeptide D	69241	Polr2d	5433	POLR2D
gnf1m09593_a_at	replication factor C (activator 1) 4	106344	Rfc4	5984	RFC4
gnf1m09603_s_at	TSR1, 20S rRNA accumulation, homolog (yeast)	104662	Tsr1	55720	TSR1
gnf1m09880_s_at	poly A binding protein, cytoplasmic 4	230721	Pabpc4	8761	PABPC4
gnf1m09911_a_at	regulator of chromosome condensation 1	100088	Rcc1	1104	RCC1
gnf1m09958_s_at	small nuclear ribonucleoprotein polypeptide G	68011	Snrpg	6637	SNRPG
gnf1m09991_a_at	hypothetical Lipocalin-related protein and Bos/Can/Equ allergen containing protein				
gnf1m10027_s_at	small nuclear ribonucleoprotein polypeptide A	53607	Snrpa	6626	SNRPA
gnf1m10054_s_at	peptidylprolyl isomerase (cyclophilin)-like 1	68816	Ppil1	51645	PPIL1
gnf1m10117_a_at	nucleoporin 107	103468	Nup107	57122	NUP107
gnf1m10118_s_at	small nuclear ribonucleoprotein D1	20641	Snrpd1	6632	SNRPD1
gnf1m10177_s_at	peptidylprolyl isomerase D (cyclophilin D)	67738	Ppid	5481	PPID
gnf1m10201_a_at	eukaryotic translation initiation factor 1A	13664	Eif1a	9086	EIF1AY

Adult Down

gnf1m10238_a_at	M phase phosphoprotein 6	68533	Mphosph6	10200	MPHOSPH6
gnf1m10294_a_at	cirrhosis, autosomal recessive 1A (human)	21771	Cirh1a	84916	CIRH1A
gnf1m10298_s_at	cell division cycle 34 homolog (S. cerevisiae)	216150	Cdc34	997	CDC34
gnf1m10310_s_at	polypyrimidine tract binding protein 1	19205	Ptbp1	5725	PTBP1
gnf1m10405_a_at	ENSMUST00000089953 transcript (in rel.37.34e)				
gnf1m10481_s_at	acidic nuclear phosphoprotein 32 family, member B	67628	Anp32b	10541	ANP32B
gnf1m10514_a_at	EBNA1 binding protein 2	69072	Ebna1bp2	10969	EBNA1BP2
gnf1m10574_a_at	spermidine synthase	20810	Srm	6723	SRM
gnf1m10578_a_at	cyclin-dependent kinase 2	12566	Cdk2	1017	CDK2
gnf1m10615_a_at	ajuba	16475	Jub	84962	AJUBA
gnf1m10616_a_at	uracil DNA glycosylase	22256	Ung	7374	UNG
gnf1m10750_a_at	nucleolar protein family A, member 1 (H/ACA small nucleolar RNPs)	68147	Nola1	54433	GAR1
gnf1m10764_a_at	tripartite motif-containing 59	66949	Trim59	286827	TRIM59
gnf1m10766_a_at	GTP binding protein 4	69237	Gtpbp4	23560	GTPBP4
gnf1m10793_a_at	Zwilch, kinetochore associated, homolog (Drosophila)	68014	Zwilch	55055	ZWILCH
gnf1m10814_s_at	RAD54 like (S. cerevisiae)	19366	Rad54l	8438	RAD54L
gnf1m10844_a_at	Luc7 homolog (S. cerevisiae)-like	66978	Luc7l	55692	LUC7L
gnf1m10846_a_at	spindle pole body component 25 homolog (S. cerevisiae)	66442	Spbc25	57405	SPC25
gnf1m10894_a_at	RIKEN cDNA 2810004N23 gene	66523	2810004N23Rik	128061	C1orf131
gnf1m10945_a_at	inosine 5'-phosphate dehydrogenase 2	23918	Impdh2	3615	IMPDH2
gnf1m10974_a_at	basic leucine zipper and W2 domains 2	66912	Bzw2	28969	BZW2
gnf1m11089_x_at	karyopherin (importin) alpha 2	16647	Kpna2	3838	KPNA2
gnf1m11183_a_at	barrier to autointegration factor 1	23825	Banf1	8815	BANF1
gnf1m11185_a_at	acidic ribosomal phosphoprotein P0	11837	Arbp	6175	RPLP0
gnf1m11192_a_at	cell division cycle associated 3	14793	Cdca3	83461	CDCA3
gnf1m11201_a_at	acidic nuclear phosphoprotein 32 family, member B	67628	Anp32b	10541	ANP32B
gnf1m11222_a_at	F-box only protein 5	67141	Fbxo5	26271	FBXO5
gnf1m11227_a_at	RAD51 homolog (S. cerevisiae)	19361	Rad51	5888	RAD51
gnf1m11244_a_at	cyclin-dependent kinase 4	12567	Cdk4	1019	CDK4
gnf1m11245_a_at	maternal embryonic leucine zipper kinase	17279	Melk	9833	MELK
gnf1m11246_a_at	deoxythymidylate kinase	21915	Dtymk	1841	DTYMK
gnf1m11247_a_at	tubulin, beta 5	22154	Tubb5	203068	TUBB
gnf1m11258_x_at	similar to High mobility group protein 2 (HMG-2)	433799	LOC433799		
gnf1m11261_a_at	cysteine and glycine-rich protein 2	13008	Csrp2	1466	CSRP2

Adult Down

gnf1m11271_a_at	RIKEN cDNA 2810037C03 gene	109145	2810037C03Rik	84296	GINS4
gnf1m11288_at	heterogeneous nuclear ribonucleoprotein A/B	15384	Hnrpab	3182	HNRNPAB
gnf1m11296_x_at	small nuclear ribonucleoprotein polypeptide A	53607	Snrpa	6626	SNRPA
gnf1m11301_s_at	insulin-like growth factor 2, binding protein 1	140486	Igf2bp1	10642	IGF2BP1
gnf1m11342_a_at	transforming, acidic coiled-coil containing protein 3	21335	Tacc3	10460	TACC3
gnf1m11369_a_at	protein arginine N-methyltransferase 5	27374	Prmt5	10419	PRMT5
gnf1m11426_a_at	RIKEN cDNA 2810430M08 gene	67223	2810430M08Rik	51018	RRP15
gnf1m11442_a_at	sorting nexin 5	69178	Snx5	27131	SNX5
gnf1m11443_a_at	retroviral integration site 2	67177	Ris2	81620	CDT1
gnf1m11462_a_at	COX4 neighbor	18117	Cox4nb	10328	COX4NB
gnf1m11586_a_at	DnaJ (Hsp40) homolog, subfamily C, member 9	108671	Dnajc9	23234	DNAJC9
gnf1m11622_a_at	ribosomal protein L13a	22121	Rpl13a	23521	RPL13A
gnf1m11645_a_at	dCMP deaminase	320685	Dctd	1635	DCTD
gnf1m11654_at	hepatoma-derived growth factor	15191	Hdgf	3068	HDGF
gnf1m11668_a_at	chaperonin subunit 7 (eta)	12468	Cct7	10574	CCT7
gnf1m11693_a_at	SNAP-associated protein	20615	Snapap	23557	SNAPIN
gnf1m11694_a_at	cyclin-dependent kinase inhibitor 1C (P57)	12577	Cdkn1c	1028	CDKN1C
gnf1m11724_a_at	nucleolin	17975	Ncl		
gnf1m11728_a_at	spindle pole body component 24 homolog (S. cerevisiae)	67629	Spbc24	147841	SPC24
gnf1m11737_a_at	interleukin enhancer binding factor 2	67781	Ilf2	3608	ILF2
gnf1m11738_a_at	replication protein A2	19891	Rpa2	6118	RPA2
gnf1m11756_a_at	minichromosome maintenance deficient 6 (MIS5 homolog, S. pombe) (S. cerevisiae)	17219	Mcm6	4175	MCM6
gnf1m11861_a_at	minichromosome maintenance deficient 5, cell division cycle 46 (S. cerevisiae)	17218	Mcm5	4174	MCM5
gnf1m11874_s_at	small nuclear ribonucleoprotein D3	67332	Snrpd3	6634	SNRPD3
gnf1m11960_a_at	cyclin E1	12447	Ccne1	898	CCNE1
gnf1m11967_at	chromatin assembly factor 1, subunit A (p150)	27221	Chaf1a	10036	CHAF1A
gnf1m11968_a_at	chromatin assembly factor 1, subunit A (p150)	27221	Chaf1a	10036	CHAF1A
gnf1m12031_a_at	KDEL (Lys-Asp-Glu-Leu) containing 1	72050	Kdelc1	79070	KDELC1
gnf1m12093_a_at	minichromosome maintenance deficient 4 homolog (S. cerevisiae)	17217	Mcm4	4173	MCM4
gnf1m12541_a_at	Mki67 (FHA domain) interacting nucleolar phosphoprotein	67949	Mki67ip	84365	MKI67IP
gnf1m12660_a_at	ribosomal protein S6 kinase polypeptide 6	67071	Rps6ka6	27330	RPS6KA6

Adult Down

gnf1m12685_a_at	MAD2 (mitotic arrest deficient, homolog)-like 1 (yeast)	56150	Mad2l1	4085	MAD2L1
gnf1m12825_s_at	protogenin homolog (Gallus gallus)	235472	Prtg	283659	PRTG
gnf1m12827_a_at	insulin-like growth factor 2, binding protein 1	140486	Igf2bp1	10642	IGF2BP1
gnf1m12839_a_at	dephospho-CoA kinase domain containing	68087	Dcakd	79877	DCAKD
gnf1m12870_a_at	FK506 binding protein 4	14228	Fkbp4	2288	FKBP4
gnf1m12873_a_at	RNA binding motif protein, X chromosome	19655	Rbmx	27316	RBMX
gnf1m12877_a_at	MYB binding protein (P160) 1a	18432	Mybbp1a	10514	MYBBP1A
gnf1m12902_a_at	fidgetin-like 1	60530	Fignl1	63979	FIGNL1
gnf1m12950_a_at	nucleolar complex associated 4 homolog (S. cerevisiae)	100608	Noc4l	79050	NOC4L
gnf1m13110_a_at	kinesin family member 2C	73804	Kif2c	11004	KIF2C
gnf1m13120_a_at	eukaryotic translation initiation factor 4A1	13681	Eif4a1	1973	EIF4A1
gnf1m13125_a_at	nudix (nucleoside diphosphate linked moiety X)-type motif 5	53893	Nudt5	11164	NUDT5
gnf1m13133_a_at	general transcription factor II E, polypeptide 2 (beta subunit)	68153	Gtf2e2	2961	GTF2E2
gnf1m13155_a_at	replication protein A3	68240	Rpa3	6119	RPA3
gnf1m13161_a_at	cyclin B1	268697	Ccnb1		
gnf1m13251_a_at	Bloom syndrome homolog (human)	12144	Blm	641	BLM
gnf1m13254_a_at	SMC2 structural maintenance of chromosomes 2-like 1 (yeast)	14211	Smc2l1	10592	SMC2
gnf1m13279_s_at	THO complex 4	21681	Thoc4	10189	ALYREF
gnf1m13304_a_at	exosome component 7	66446	Exosc7	23016	EXOSC7
gnf1m13388_a_at	solute carrier family 16 (monocarboxylic acid transporters), member 3	80879	Slc16a3	9123	SLC16A3
gnf1m13465_a_at	C-terminal binding protein 2	13017	Ctbp2	1488	CTBP2
gnf1m13587_a_at	aurora kinase A	20878	Aurka	6790	AURKA
gnf1m13610_a_at	cDNA sequence BC003885	225215	BC003885	51187	RSL24D1
gnf1m13629_a_at	replication factor C (activator 1) 3	69263	Rfc3	5983	RFC3
gnf1m13853_a_at	RAN binding protein 5	70572	Ranbp5	3843	IPO5
gnf1m15316_at	thymopoietin	21917	Tmpo	7112	TMPO
gnf1m15317_at	RIKEN cDNA 2610206G21 gene	72491	2610206G21Rik		
gnf1m15330_a_at	DNA segment, Chr 19, Brigham & Women's Genetics 1357 expressed	52874	D19Bwg1357e	9933	KIAA0020
gnf1m15371_s_at	myelin basic protein expression factor 2, repressor	17876	Myef2	50804	MYEF2
gnf1m15726_a_at	RNA binding motif protein 13	67920	Rbm13	84549	MAK16
gnf1m15837_x_at	hypothetical protein LOC630153	630153	LOC630153		
gnf1m15888_at	chloride channel 5	12728	Clcn5	1184	CLCN5
gnf1m16029_at	enolase 1, alpha non-neuron				

Adult Down

gnf1m16048_a_at	RIKEN cDNA C330012H03 gene	319765	C330012H03Rik	10644	IGF2BP2
gnf1m16085_at	Mouse clone IMAGE:3493906, mRNA, partial cds /cds=UNKNOWN /gb=BC004016 /gi=13278408 /len=1036 gnl|UG|Mm#S2006337				
gnf1m16095_s_at	RIKEN cDNA 2410019A14 gene	229937	2410019A14Rik	54680	ZNHIT6
gnf1m16176_a_at	septin 11	52398	'Sept11	55752	40431
gnf1m16177_a_at	peroxidasin homolog (Drosophila)	69675	Pxdn	7837	PXDN
gnf1m16238_a_at	coiled-coil domain containing 58	381045	Ccdc58	131076	CCDC58
gnf1m16239_a_at	methylthioadenosine phosphorylase	66902	Mtap	4507	MTAP
gnf1m16250_at	SET translocation	56086	Set	6418	SET
gnf1m16281_a_at	golgi associated, gamma adaptin ear containing, ARF binding protein 2	74105	Gga2	23062	GGA2
gnf1m16370_a_at	RIKEN cDNA 5730507H05 gene	54392	5730507H05Rik		
gnf1m16487_a_at	aurora kinase B	20877	Aurkb	9212	AURKB
gnf1m16626_a_at	antigen identified by monoclonal antibody Ki 67	17345	Mki67	4288	MKI67
gnf1m16799_a_at	branched chain aminotransferase 1, cytosolic	12035	Bcat1	586	BCAT1
gnf1m17121_a_at	thyroid hormone receptor interactor 13	69716	Trip13	9319	TRIP13
gnf1m17337_a_at	RIKEN cDNA 2810418N01 gene	69270	2810418N01Rik	9837	GINS1
gnf1m17411_at	podocalyxin-like	27205	Podxl	5420	PODXL
gnf1m17421_a_at	polymerase (DNA directed), epsilon 2 (p59 subunit)	18974	Pole2	5427	POLE2
gnf1m17492_at	sperm associated antigen 5	54141	Spag5	10615	SPAG5
gnf1m17599_at	Chimeric clone containing F-box and WD-40 domain protein 7, archipelago and RAN, member RAS oncogene family				
gnf1m17616_a_at	nucleoporin 54	269113	Nup54	53371	NUP54
gnf1m18045_at	gb=AJ409483 Mouse RNA binding site for Dazl protein, clone bd8				
gnf1m18307_a_at	myelin protein zero-like 1	68481	Mpzl1	9019	MPZL1
gnf1m18565_a_at	RIKEN cDNA 2810026P18 gene	72655	2810026P18Rik		
gnf1m18759_a_at	RIKEN cDNA 5033413D16 gene	75957	5033413D16Rik		
gnf1m18976_a_at	E2F transcription factor 8	108961	E2f8	79733	E2F8
gnf1m19005_a_at	KH-type splicing regulatory protein	16549	Khsrp		
gnf1m19030_at	unclassifiable				
gnf1m19090_a_at	brain expressed gene 4	406217	Bex4	56271	BEX4
gnf1m20871_at	splicing factor, arginine/serine rich 9 (25 kDa)				
gnf1m21457_at	angiomotin	27494	Amot	154796	AMOT

Adult Down

gnf1m22621_at	unclassifiable				
gnf1m22622_s_at	X-linked myotubular myopathy gene 1	17772	Mtm1	4534	MTM1
gnf1m23455_s_at	DNA2 DNA replication helicase 2-like (yeast)	327762	Dna2l	1763	DNA2
gnf1m23503_at	proliferation-associated 2G4		Pa2g4		
gnf1m23789_at	enolase 1, alpha non-neuron		Eno1		
gnf1m23992_a_at	growth factor receptor bound protein 10	14783	Grb10	2887	GRB10
gnf1m24038_a_at	kinesin family member 23	71819	Kif23	9493	KIF23
gnf1m24048_a_at	transcription factor Dp 1	21781	Tfdp1	7027	TFDP1
gnf1m24084_a_at	nuclear receptor coactivator 6 interacting protein	116940	Ncoa6ip		
gnf1m24859_at	nucleolar and coiled-body phosphoprotein 1	70769	Nolc1	9221	NOLC1
gnf1m24910_at	high mobility group AT-hook 2	15364	Hmga2	8091	HMGA2
gnf1m24922_at	HEART- AND NEURAL CREST DERIVATIVES-EXPRESSED PROTEIN 2 (DECIDUUM, HEART, AUTONOMIC NERVOUS SYSTEM AND NEURAL CREST DERIVATIVES- EXPRESSED PROTEIN 2) (DHAND) homolog [Homo sapiens]				
gnf1m24965_a_at	expressed sequence AU018661				
gnf1m25088_a_at	unclassifiable				
gnf1m25171_a_at	RIKEN cDNA 6230416J20 gene	230376	6230416J20Rik	54801	HAUS6
gnf1m27092_a_at	bystin-like	53414	Bysl	705	BYSL
gnf1m27340_at	inferred: Unknown (protein for MGC:11691) [Mus musculus]				
gnf1m27401_s_at	RIKEN cDNA 2310008H09 gene	66356	2310008H09Rik	400506	C16orf88
gnf1m27432_x_at	ERYTHROID DIFFERENTIATION REGULATOR (FRAGMENT) homolog [Mus musculus]				
gnf1m27734_at	unknown EST				
gnf1m27782_at	insulin-like growth factor 2, binding protein 1	140486	Igf2bp1	10642	IGF2BP1
gnf1m28171_a_at	orthodenticle homolog 2 (Drosophila)	18424	Otx2	5015	OTX2
gnf1m28353_a_at	minichromosome maintenance deficient 3 (S. cerevisiae)	17215	Mcm3	4172	MCM3
gnf1m28417_a_at	tubulin, beta 5	22154	Tubb5	203068	TUBB
gnf1m28595_a_at	adenylate kinase 2	11637	Ak2	204	AK2
gnf1m28638_a_at	spermatogenesis associated, serine-rich 2	72572	Spats2	65244	SPATS2
gnf1m28822_a_at	replication factor C (activator 1) 5	72151	Rfc5	5985	RFC5
gnf1m28919_a_at	guanine monphosphate synthetase	229363	Gmps	8833	GMPS
gnf1m28953_at	asparagine-linked glycosylation 8 homolog (yeast, alpha-1,3-glucosyltransferase)	381903	Alg8	79053	ALG8
gnf1m29077_a_at	phosphoserine aminotransferase 1	107272	Psat1	29968	PSAT1
gnf1m29114_at	WD repeat domain 43	72515	Wdr43	23160	WDR43

Adult Down

gnf1m29189_at	RIKEN cDNA 2410044K02 gene RIKEN cDNA 2410044K02 gene mCG4377				
gnf1m29212_a_at	TPX2, microtubule-associated protein homolog (Xenopus laevis)	72119	Tpx2	22974	TPX2
gnf1m29286_a_at	carbamoyl-phosphate synthetase 2, aspartate transcarbamylase, and dihydroorotase	69719	Cad	790	CAD
gnf1m29376_a_at	M-phase phosphoprotein 1	240641	Mphosph1	9585	KIF20B
gnf1m29466_a_at	DNA segment, Chr 7, Wayne State University 128, expressed	28018	D7Wsu128e	56061	UBFD1
gnf1m29476_a_at	nucleoporin 205	70699	Nup205	23165	NUP205
gnf1m29532_a_at	structure specific recognition protein 1	20833	Ssrp1	6749	SSRP1
gnf1m29651_a_at	kinesin family member 11	16551	Kif11	3832	KIF11
gnf1m29697_at	carbohydrate (chondroitin) synthase 1	269941	Chsy1	22856	CHSY1
gnf1m29831_a_at	tropomyosin 4	326618	Tpm4		
gnf1m29894_a_at	RIKEN cDNA 2610033H07 gene	75416	2610033H07Rik	8602	NOP14
gnf1m29907_at	inferred: gb|AAH21337.1|AAH21337 (BC021337) Similar to hypothetical protein FLJ10407 [Mus musculus] mCG16076				
gnf1m29955_a_at	DNA segment, Chr 17, ERATO Doi 441, expressed	52009	D17Ertd441e	90861	HN1L
gnf1m29962_a_at	transmembrane protein with EGF-like and two follistatin-like domains 1	230157	Tmeff1	8577	TMEFF1
gnf1m30152_a_at	thymidylate kinase				
gnf1m30192_a_at	RIKEN cDNA 2610318N02 gene	70458	2610318N02Rik		
gnf1m30254_a_at	kinetochore associated 1	208628	Kntc1	9735	KNTC1
gnf1m30630_a_at	RIKEN cDNA 2610012O22 gene	69902	2610012O22Rik	51154	MRTO4
gnf1m30739_a_at	amine oxidase (flavin containing) domain 2	99982	Aof2	23028	KDM1A
gnf1m31118_a_at	DEAD (Asp-Glu-Ala-Asp) box polypeptide 39	68278	Ddx39	10212	DDX39A
gnf1m31147_s_at	nucleolar and coiled-body phosphoprotein 1	70769	Nolc1	9221	NOLC1
gnf1m31235_s_at	unclassifiable				
gnf1m32171_at	nucleoporin 155				
gnf1m32177_x_at	similar to SET protein (Phosphatase 2A inhibitor I2PP2A) (I-2PP2A) (Template activating factor I) (TAF-I) (Liver regeneration-related protein LRRGR00002)	625349	LOC625349		
gnf1m32805_x_at	similar to nuclease sensitive element binding protein 1	432601	LOC432601		
gnf1m32923_at	extra spindle poles-like 1 (S. cerevisiae)	105988	Espl1	9700	ESPL1

Adult Down

gnf1m33390_x_at	inferred: gb|AAG59811.1|AF308818_1 (AF308818) MAP2 RNA trans-acting protein MARTA1 [Rattus norvegicus] mCG125615				
gnf1m33790_at	cytoskeleton associated protein 2	80986	Ckap2	26586	CKAP2
gnf1m33909_x_at	erythroid differentiation regulator 1	170942	Erdr1		
gnf1m34205_x_at	similar to Importin alpha-2 subunit (Karyopherin alpha-2 subunit) (SRP1-alpha) (RAG cohort protein 1) (Pendulin) (Pore targeting complex 58 kDa subunit) (PTAC58) (Importin alpha P1)	545589	LOC545589		
gnf1m34258_x_at	inferred: ref|NP_058797.1| (NM_017101) peptidylprolyl isomerase A (cyclophilin A) [Rattus norvegicus] mCG8332				
gnf1m34382_x_at	similar to ribosomal protein S15a	434460	LOC434460		
gnf1m34528_at	inferred: ref|NP_000998.1| (NM_001007) ribosomal protein S4, X isoform; cell cycle gene 2; 40S ribosomal protein S4, X isoform; ribosomal protein S4X isoform; single-copy abundant mRNA [Homo sapiens] mCG1043328				
gnf1m35452_x_at	inferred: ref|NP_058846.1| (NM_017150) ribosomal protein L29 [Rattus norvegicus] mCG1032957				

E6.5 Up					
**Probe Set ID**	**Description**	**LocusLink Mouse**	**Gene Symbol Mouse**	**LocusLink Human**	**Gene Symbol Human**
gnf1m00049_a_at	laminin, gamma 1	226519	Lamc1	3915	LAMC1
gnf1m00168_a_at	adenine phosphoribosyl transferase	11821	Aprt	353	APRT
gnf1m00375_at	growth factor receptor bound protein 2-associated protein 1	14388	Gab1	2549	GAB1
gnf1m00478_a_at	keratin complex 2, basic, gene 7	110310	Krt2-7	3855	KRT7
gnf1m00512_a_at	epithelial V-like antigen 1	14012	Eva1	10205	MPZL2
gnf1m00587_a_at	RNA binding motif protein 19	74111	Rbm19	9904	RBM19
gnf1m00684_a_at	pseudouridylate synthase 7 homolog (S. cerevisiae)	78697	Pus7	54517	PUS7
gnf1m00826_a_at	aquaporin 8	11833	Aqp8	343	AQP8
gnf1m00844_a_at	solute carrier family 7 (cationic amino acid transporter, y+ system), member 3	11989	Slc7a3	84889	SLC7A3
gnf1m00896_a_at	carbonic anhydrase 4	12351	Car4	762	CA4
gnf1m00944_a_at	Cbp/p300-interacting transactivator with Glu/Asp-rich carboxy-terminal domain 1	12705	Cited1	4435	CITED1
gnf1m00999_a_at	cytochrome P450, family 26, subfamily a, polypeptide 1	13082	Cyp26a1	1592	CYP26A1
gnf1m01170_a_at	GATA binding protein 2	14461	Gata2	2624	GATA2
gnf1m01186_a_at	gap junction membrane channel protein beta 3	14620	Gjb3	2707	GJB3
gnf1m01230_a_at	heart and neural crest derivatives expressed transcript 1	15110	Hand1	9421	HAND1
gnf1m01364_a_at	keratin complex 1, acidic, gene 19	16669	Krt1-19		
gnf1m01370_a_at	laminin, alpha 1	16772	Lama1	284217	LAMA1
gnf1m01503_a_at	N-myristoyltransferase 2	18108	Nmt2	9397	NMT2
gnf1m01514_a_at	nucleoplasmin 3	18150	Npm3	10360	NPM3
gnf1m01566_a_at	reproductive homeobox 5	18617	Rhox5		
gnf1m01626_a_at	protease, serine, 12 neurotrypsin (motopsin)	19142	Prss12	8492	PRSS12
gnf1m01726_a_at	scinderin	20259	Scin	85477	SCIN
gnf1m01777_a_at	sterol O-acyltransferase 1	20652	Soat1	6646	SOAT1
gnf1m01790_a_at	serine peptidase inhibitor, Kazal type 3	20730	Spink3	6690	SPINK1
gnf1m01820_a_at	brachyury	20997	T	6862	T
gnf1m01834_a_at	transcription factor AP-2, gamma	21420	Tcfap2c	7022	TFAP2C
gnf1m01893_a_at	pleckstrin homology-like domain, family A, member 2	22113	Phlda2	7262	PHLDA2
gnf1m01916_a_at	uridine phosphorylase 1	22271	Upp1	7378	UPP1
gnf1m01922_a_at	undifferentiated embryonic cell transcription factor 1	22286	Utf1	8433	UTF1

E6.5 Up

gnf1m01956_a_at	zinc finger protein 42	22702	Zfp42	132625	ZFP42
gnf1m01983_at	adrenomedullin	11535	Adm	133	ADM
gnf1m02023_a_at	apolipoprotein A-I	11806	Apoa1	335	APOA1
gnf1m02194_a_at	DNA methyltransferase 3B	13436	Dnmt3b	1789	DNMT3B
gnf1m02205_at	decidual/trophoblast prolactin-related protein	13529	Dtprp		
gnf1m02219_a_at	E74-like factor 5	13711	Elf5	2001	ELF5
gnf1m02264_a_at	fos-like antigen 1	14283	Fosl1	8061	FOSL1
gnf1m02285_a_at	glycoprotein galactosyltransferase alpha 1, 3	14594	Ggta1		
gnf1m02304_a_at	podoplanin	14726	Pdpn	10630	PDPN
gnf1m02350_a_at	histone deacetylase 6	15185	Hdac6	10013	HDAC6
gnf1m02368_a_at	heme oxygenase (decycling) 1	15368	Hmox1	3162	HMOX1
gnf1m02413_a_at	interleukin 1 receptor, type II	16178	Il1r2	7850	IL1R2
gnf1m02544_a_at	myelocytomatosis oncogene	17869	Myc	4609	MYC
gnf1m02561_a_at	NIMA (never in mitosis gene a)-related expressed kinase 2	18005	Nek2	4751	NEK2
gnf1m02650_x_at	proliferin 2	18812	Plf2		
gnf1m02673_a_at	prolactin-like protein A	19110	Prlpa		
gnf1m02674_a_at	prolactin-like protein B	19111	Prlpb		
gnf1m02746_a_at	secretin	20287	Sct	6343	SCT
gnf1m02759_a_at	sema domain, immunoglobulin domain (Ig), short basic domain, secreted, (semaphorin) 3E	20349	Sema3e	9723	SEMA3E
gnf1m02789_a_at	solute carrier family 7 (cationic amino acid transporter, y+ system), member 7	20540	Slc7a7	9056	SLC7A7
gnf1m02811_a_at	serine (or cysteine) peptidase inhibitor, clade B, member 9b	20706	Serpinb9b		
gnf1m02812_a_at	serine (or cysteine) peptidase inhibitor, clade B, member 9e	20710	Serpinb9e		
gnf1m02813_s_at	serine (or cysteine) peptidase inhibitor, clade B, member 9e	20710	Serpinb9e		
gnf1m02844_a_at	syndecan 4	20971	Sdc4	6385	SDC4
gnf1m02855_a_at	teratocarcinoma-derived growth factor	21667	Tdgf1	6997	TDGF1
gnf1m02891_at	tumor rejection antigen P1A	22037	Trap1a		
gnf1m02930_a_at	X-linked lymphocyte-regulated 3A	22445	Xlr3a		
gnf1m03034_a_at	glucosamine-6-phosphate deaminase 1	26384	Gnpda1	10007	GNPDA1
gnf1m03202_a_at	POU domain, class 5, transcription factor 1	18999	Pou5f1	5460	POU5F1
gnf1m03276_a_at	prolactin-like protein I	27372	Prlpi		
gnf1m03349_a_at	ring finger protein 12	19820	Rnf12	51132	RLIM

E6.5 Up

gnf1m03539_a_at	myeloid-associated differentiation marker	50918	Myadm	91663	MYADM
gnf1m03568_a_at	polymerase (DNA directed), gamma	18975	Polg	5428	POLG
gnf1m03633_a_at	apolipoprotein M	55938	Apom	55937	APOM
gnf1m03726_a_at	placental specific protein 1	56096	Plac1	10761	PLAC1
gnf1m03727_a_at	pore forming protein-like	56093	Pfpl		
gnf1m03882_a_at	prolactin-like protein M	56635	Prlpm		
gnf1m04020_a_at	reproductive homeobox 4B	57737	Rhox4b		
gnf1m04097_a_at	phorbol-12-myristate-13-acetate-induced protein 1	58801	Pmaip1		
gnf1m04205_a_at	cathepsin Z	64138	Ctsz	1522	CTSZ
gnf1m04452_s_at	developmental pluripotency associated 5	434423	Dppa5	340168	DPPA5
gnf1m04505_a_at	eukaryotic translation elongation factor 1 epsilon 1	66143	Eef1e1	9521	EEF1E1
gnf1m04531_a_at	zinc finger, DHHC domain containing 12	66220	Zdhhc12	84885	ZDHHC12
gnf1m04726_a_at	related RAS viral (r-ras) oncogene homolog 2	66922	Rras2	22800	RRAS2
gnf1m04855_a_at	plakophilin 2	67451	Pkp2	5318	PKP2
gnf1m04938_a_at	jagunal homolog 1 (Drosophila)	67767	Jagn1	84522	JAGN1
gnf1m04979_a_at	tubulin, beta 6	67951	Tubb6	84617	TUBB6
gnf1m05119_a_at	endothelial cell-specific adhesion molecule	69524	Esam1	90952	ESAM
gnf1m05231_s_at	synaptonemal complex central element protein 2	71846	Syce2	256126	SYCE2
gnf1m05291_a_at	cullin 4B	72584	Cul4b	8450	CUL4B
gnf1m05303_a_at	anillin, actin binding protein (scraps homolog, Drosophila)	68743	Anln	54443	ANLN
gnf1m05329_a_at	testis expressed gene 19	73679	Tex19		
gnf1m05569_a_at	nuclear factor of kappa light polypeptide gene enhancer in B-cells inhibitor, zeta	80859	Nfkbiz	64332	NFKBIZ
gnf1m05759_a_at	amnionless	93835	Amn	81693	AMN
gnf1m05811_a_at	kinesin family member C1	16580	Kifc1	3833	KIFC1
gnf1m06008_a_at	protease, serine, 8 (prostasin)	76560	Prss8	5652	PRSS8
gnf1m06039_a_at	transmembrane protein 97	69071	Tmem97	27346	TMEM97
gnf1m06251_a_at	glycine decarboxylase	104174	Gldc	2731	GLDC
gnf1m06256_s_at	proviral integration site 2	18715	Pim2	11040	PIM2
gnf1m06312_a_at	placenta-specific 8	231507	Plac8	51316	PLAC8
gnf1m06363_a_at	Wilms tumor homolog	22431	Wt1	7490	WT1
gnf1m06473_a_at	eukaryotic translation initiation factor 2B, subunit 1 (alpha)	209354	Eif2b1	1967	EIF2B1
gnf1m06596_a_at	RNA, U3 small nucleolar interacting protein 2	27966	Rnu3ip2	9136	RRP9
gnf1m07453_a_at	claudin 6	54419	Cldn6	9074	CLDN6

E6.5 Up

gnf1m07636_a_at	RNA binding protein with multiple splicing 2	71973	Rbpms2	348093	RBPMS2
gnf1m08135_a_at	solute carrier family 13 (sodium/sulfate symporters), member 4	243755	Slc13a4	26266	SLC13A4
gnf1m08533_a_at	expressed sequence AI429152	98956	AI429152	55226	NAT10
gnf1m08880_at	matrix metallopeptidase 1a (interstitial collagenase)	83995	Mmp1a	4312	MMP1
gnf1m08881_a_at	matrix metallopeptidase 1a (interstitial collagenase)	83995	Mmp1a	4312	MMP1
gnf1m09212_s_at	RIKEN cDNA 1110033J19 gene	66184	1110033J19Rik		
gnf1m09263_a_at	amyloid beta precursor protein (cytoplasmic tail) binding protein 2	66884	Appbp2	10513	APPBP2
gnf1m09273_s_at	syndecan 1	20969	Sdc1	6382	SDC1
gnf1m09297_s_at	non imprinted in Prader-Willi/Angelman syndrome 2 homolog (human)	93790	Nipa2	81614	NIPA2
gnf1m09346_s_at	proliferin	18811	Plf		
gnf1m09355_s_at	developmental pluripotency associated 5	434423	Dppa5	340168	DPPA5
gnf1m09428_s_at	developmental pluripotency associated 5	434423	Dppa5	340168	DPPA5
gnf1m09471_a_at	cathepsin 7	56092	Cts7		
gnf1m09669_a_at	RIKEN cDNA 1600029D21 gene	76509	1600029D21Rik		
gnf1m09746_a_at	midasin homolog (yeast)	100019	Mdn1	23195	MDN1
gnf1m09844_a_at	chloride intracellular channel 6	209195	Clic6	54102	CLIC6
gnf1m10011_a_at	N-acetylneuraminate pyruvate lyase	74091	Npl	80896	NPL
gnf1m10060_s_at	developmental pluripotency associated 5	434423	Dppa5	340168	DPPA5
gnf1m10161_s_at	WD repeat domain 12	57750	Wdr12	55759	WDR12
gnf1m10185_a_at	tetraspanin 4	64540	Tspan4	7106	TSPAN4
gnf1m10261_a_at	DNA (cytosine-5-)-methyltransferase 3-like	54427	Dnmt3l	29947	DNMT3L
gnf1m10359_a_at	chorionic somatomammotropin hormone 1	18775	Csh1		
gnf1m10560_at	GM2 ganglioside activator protein	14667	Gm2a	2760	GM2A
gnf1m10648_a_at	parathyroid hormone receptor 1	19228	Pthr1	5745	PTH1R
gnf1m10709_a_at	tryptophan 2,3-dioxygenase	56720	Tdo2	6999	TDO2
gnf1m10768_a_at	apolipoprotein C-II	11813	Apoc2	344	APOC2
gnf1m10812_a_at	protein C receptor, endothelial	19124	Procr	10544	PROCR
gnf1m10924_a_at	cystatin B	13014	Cstb	1476	CSTB
gnf1m10969_a_at	natriuretic peptide precursor type B	18158	Nppb		
gnf1m11019_at	matrix metallopeptidase 9	17395	Mmp9	4318	MMP9
gnf1m11071_a_at	serine (or cysteine) peptidase inhibitor, clade B, member 9f	20709	Serpinb9f		
gnf1m11074_a_at	cytotoxic T lymphocyte-associated protein 2 alpha	13024	Ctla2a		

E6.5 Up

gnf1m11075_s_at	cytotoxic T lymphocyte-associated protein 2 alpha	13024	Ctla2a		
gnf1m11076_a_at	DNA segment, Chr 13, Wayne State University 14, expressed	28078	D13Wsu14e		
gnf1m11078_a_at	prolactin-like protein C 2	67310	Prlpc2		
gnf1m11186_a_at	ATPase family, AAA domain containing 3A	108888	Atad3a	55210	ATAD3A
gnf1m11223_a_at	galactokinase 1	14635	Galk1	2584	GALK1
gnf1m11450_a_at	prostaglandin I2 (prostacyclin) synthase	19223	Ptgis	5740	PTGIS
gnf1m11560_a_at	embryonal Fyn-associated substrate	13644	Efs	10278	EFS
gnf1m11631_at	L-threonine dehydrogenase	58865	Tdh		
gnf1m11632_s_at	similar to L-threonine dehydrogenase	433463	LOC433463		
gnf1m11821_a_at	interferon alpha responsive gene	64164	Ifrg15		
gnf1m11830_a_at	microrchidia 4	75746	Morc4	79710	MORC4
gnf1m11857_a_at	serine (or cysteine) peptidase inhibitor, clade B, member 6b	20708	Serpinb6b		
gnf1m12181_a_at	embigin	13723	Emb	133418	EMB
gnf1m12679_a_at	gap junction membrane channel protein beta 5	14622	Gjb5	2709	GJB5
gnf1m12766_a_at	transmembrane protein 37	170706	Tmem37	140738	TMEM37
gnf1m12858_a_at	guanine nucleotide binding protein (G protein), beta polypeptide 2 like 1	14694	Gnb2l1	10399	GNB2L1
gnf1m12983_a_at	peter pan homolog (Drosophila)	235036	Ppan	5032	P2RY11
gnf1m13029_a_at	follistatin-like 3	83554	Fstl3	10272	FSTL3
gnf1m13474_a_at	translocating chain-associating membrane protein 2	170829	Tram2	9697	TRAM2
gnf1m13480_a_at	nuclear RNA export factor 7	170722	Nxf7	56001	NXF2
gnf1m13579_a_at	RIKEN cDNA 2310031A18 gene	69627	2310031A18Rik	375061	FAM89A
gnf1m15327_a_at	procollagen, type IV, alpha 2	12827	Col4a2	1284	COL4A2
gnf1m15341_at	RIKEN cDNA 8430417A20 gene	74516	8430417A20Rik		
gnf1m15737_s_at	Mus musculus, clone IMAGE:3983821				
gnf1m15786_a_at	achaete-scute complex homolog-like 2 (Drosophila)	17173	Ascl2	430	ASCL2
gnf1m15831_x_at	Mouse clone IMAGE:4206769, mRNA /cds=UNKNOWN /gb=BC026940 /gi=20072489 /len=2963 gnl|UG|Mm#S2581369				
gnf1m15930_at	cytotoxic T lymphocyte-associated protein 2 beta				
gnf1m16258_at	hypothetical protein				
gnf1m16368_at	RIKEN cDNA 2410003J06 gene	71967	2410003J06Rik	139604	MAGEB16
gnf1m16840_at	tumor rejection antigen gp96				

E6.5 Up

gnf1m16953_a_at	RIKEN cDNA 3830417A13 gene	70696	3830417A13Rik		
gnf1m17166_a_at	procollagen, type IV, alpha 1	12826	Col4a1	1282	COL4A1
gnf1m17412_a_at	a disintegrin and metallopeptidase domain 19 (meltrin beta)	11492	Adam19	8728	ADAM19
gnf1m17917_at	gb=BC027702 Mouse clone IMAGE:1265476, mRNA				
gnf1m18381_a_at	RIKEN cDNA 2310005L22 gene	69471	2310005L22Rik		
gnf1m18947_s_at	eukaryotic translation initiation factor 3, subunit 1 alpha	78655	Eif3s1	8669	EIF3J
gnf1m21118_at	CEA-related cell adhesion molecule 15	101434	Ceacam15		
gnf1m21620_at	fragile X mental retardation 1 neighbor	207854	Fmr1nb	158521	FMR1NB
gnf1m23202_at	RIKEN cDNA 1810043K16 gene	108726	1810043K16Rik		
gnf1m23421_a_at	Nik related kinase				
gnf1m25433_a_at	dystroglycan 1	13138	Dag1	1605	DAG1
gnf1m25627_a_at	WD repeat domain 36	225348	Wdr36		
gnf1m26996_at	tripartite motif protein 27		Trim27		
gnf1m27314_at	follistatin				
gnf1m28522_a_at	phospholipase A2, group V	18784	Pla2g5	5322	PLA2G5
gnf1m28617_at	disabled homolog 2 (Drosophila)	13132	Dab2	1601	DAB2
gnf1m29219_a_at	kelch-like 21 (Drosophila)	242785	Klhl21	9903	KLHL21
gnf1m29348_a_at	laminin B1 subunit 1	16777	Lamb1-1	3912	LAMB1
gnf1m29351_a_at	carboxypeptidase M	70574	Cpm	1368	CPM
gnf1m29385_s_at	eomesodermin homolog (Xenopus laevis)	13813	Eomes	8320	EOMES
gnf1m29603_a_at	metallothionein 2	17750	Mt2	4502	MT2A
gnf1m29873_a_at	retinol binding protein 4, plasma	19662	Rbp4	5950	RBP4
gnf1m30139_a_at	alpha-2-macroglobulin	232345	A2m	2	A2M
gnf1m30158_a_at	Hypothetical BTB/POZ domain and hypothetical Kelch repeat containing protein homolog [Mus musculus]				
gnf1m30197_at	low density lipoprotein receptor-related protein 2	14725	Lrp2	4036	LRP2
gnf1m30648_a_at	RIKEN cDNA 2410008K03 gene	71962	2410008K03Rik	652968	GATSL3
gnf1m30737_a_at	RIKEN cDNA 6030465E24 gene	214585	6030465E24Rik		
gnf1m30764_s_at	stratifin		Sfn		
gnf1m30811_at	gene model 719, (NCBI)	279706	Gm719		
gnf1m30986_a_at	G protein-coupled receptor 116	224792	Gpr116	221395	GPR116
gnf1m31057_a_at	cubilin (intrinsic factor-cobalamin receptor)	65969	Cubn	8029	CUBN
gnf1m31430_at	kinesin family member 26A	238403	Kif26a		

E6.5 Up

gnf1m32088_at	inferred: ref|NP_036094.1| (NM_011964) pregnancy specific glycoprotein 19; carcinoembryonic antigen 4 [Mus musculus] mCG3735				
gnf1m33092_at	apolipoprotein B	238055	Apob	338	APOB
gnf1m33146_at	expressed sequence AA408556	107094	AA408556	23223	RRP12

E6.5 Down					
**Probe Set ID**	**Description**	**LocusLink Mouse**	**Gene Symbol Mouse**	**LocusLink Human**	**Gene Symbol Human**
gnf1m00326_s_at	LETM1 domain containing 1	68614	Letmd1	25875	LETMD1
gnf1m00490_a_at	Notch gene homolog 1 (Drosophila)	18128	Notch1	4851	NOTCH1
gnf1m00782_a_at	adenylate cyclase 6	11512	Adcy6	112	ADCY6
gnf1m00824_a_at	amyloid beta (A4) precursor protein	11820	App	351	APP
gnf1m03883_a_at	GTPase activating RANGAP domain-like 1	56784	Garnl1	253959	RALGAPA1
gnf1m04612_a_at	CREBBP/EP300 inhibitory protein 1	58521	Cri1	23741	EID1
gnf1m06670_a_at	ankyrin 3, epithelial	11735	Ank3	288	ANK3
gnf1m06719_a_at	expressed sequence AW146242	232023	AW146242	81552	VOPP1
gnf1m10847_a_at	SH3-binding domain glutamic acid-rich protein like	56726	Sh3bgrl	6451	SH3BGRL
gnf1m10853_a_at	G protein-coupled receptor 56	14766	Gpr56	9289	GPR56
gnf1m11189_a_at	annexin A6	11749	Anxa6	309	ANXA6
gnf1m13454_at	RIKEN cDNA 1700020I14 gene	66602	1700020I14Rik		
gnf1m13466_a_at	serologically defined colon cancer antigen 33	110796	Sdccag33	10194	TSHZ1
gnf1m15342_s_at	N-deacetylase/N-sulfotransferase (heparan glucosaminyl) 1	15531	Ndst1	3340	NDST1
gnf1m16289_a_at	heterochromatin protein 1, binding protein 3	15441	Hp1bp3	50809	HP1BP3
gnf1m16411_at	homeodomain interacting protein kinase 2	15258	Hipk2	28996	HIPK2
gnf1m16695_a_at	kinesin family member 1B	16561	Kif1b	23095	KIF1B
gnf1m16997_a_at	insulin-like growth factor binding protein 5	16011	Igfbp5	3488	IGFBP5
gnf1m17097_at	ornithine decarboxylase antizyme 2	18247	Oaz2	4947	OAZ2
gnf1m17451_a_at	attractin like 1	226255	Atrnl1	26033	ATRNL1
gnf1m18989_at	Kruppel-like factor 3 (basic)	16599	Klf3	51274	KLF3
gnf1m22543_a_at	HEG homolog 1 (zebrafish)	77446	Heg1	57493	HEG1
gnf1m22836_a_at	RIKEN cDNA 9530068E07 gene	213673	9530068E07Rik	56951	C5orf15
gnf1m22986_a_at	zinc finger, MYND domain containing 11	66505	Zmynd11	10771	ZMYND11
gnf1m23112_a_at	neuropilin				
gnf1m24964_s_at	expressed sequence C78339	97863	C78339	51439	FAM8A1
gnf1m27672_a_at	Musashi homolog 2 (Drosophila)	76626	Msi2h	124540	MSI2
gnf1m28716_a_at	kelch repeat and BTB (POZ) domain containing 7	211255	Kbtbd7	84078	KBTBD7
gnf1m28756_a_at	tubulin, beta 2				
gnf1m29182_at	praja 2, RING-H2 motif containing	224938	Pja2	9867	PJA2
gnf1m29766_a_at	RIKEN cDNA 2310047A01 gene	71918	2310047A01Rik	219654	ZCCHC24
gnf1m30905_a_at	metastasis suppressor 1	211401	Mtss1	9788	MTSS1
gnf1m31150_a_at	kelch-like 5 (Drosophila)	71778	Klhl5		

E6.5 Down

gnf1m32160_at	inferred: ref|NP_006132.1| (NM_006141) dynein, cytoplasmic, light intermediate polypeptide 2 [Homo sapiens] mCG21553

Suppl Table-2

Significantly altered gene networks represented in the
E6.5 gene expression signature

	Altered Functional Network	Genes Regulated	p-value
**E6.5 Up**	Tissue Morphology	APOB,CITED1,COL4A1,CUL4B,DAB2,DAG1,Elf5,GAB1,GATA,HAND1,MYC,PROCR,T,TDGF1	2.34E-10
	Cellular Growth and Proliferation	HMOX1,LAMA1,MYC,SDC1,TUBB6	5.04E-10
	Embryonic Development	ADM,CITED1,COL4A1,DNMT3L,FOSL1,GAB1,GATA2,GJB3, PHLDA2,UTF1	3.90E-09
	Cellular Movement	A2M,ADAM19,ADM,APOA1,APOB,AQP8,COL4A1,COL4A2,CTSZ,CYP26A1,DAB2,DAG1,EFS,EOMES,FOSL1,GAB1,GATA2,GNB2L1,GPR116,HDAC6,HMOX1,LAMA1,LAMC1,MMP1,MMP9,MYADM,MYC,NFKBIZ,PDPN,PHLDA2,PLA2G5,PLAC1,POU5F1,PROCR,RRAS2,SDC1,SDC4,SEMA3E,SFN,TDGF1	7.05E-09
**E6.5 Down**	Nervous System Development and Function	APP,KIF1B,NDST1	3.04E-06
	Cellular Compromise, Neurological Disease, Organismal Injury and Abnormalities	APP,KIF1B	3.55E-05
